# Hippocampal long‐term synaptic depression and memory deficits induced in early amyloidopathy are prevented by enhancing G‐protein‐gated inwardly rectifying potassium channel activity

**DOI:** 10.1111/jnc.14946

**Published:** 2020-01-30

**Authors:** Irene Sánchez‐Rodríguez, Souhail Djebari, Sara Temprano‐Carazo, David Vega‐Avelaira, Raquel Jiménez‐Herrera, Guillermo Iborra‐Lázaro, Javier Yajeya, Lydia Jiménez‐Díaz, Juan D. Navarro‐López

**Affiliations:** ^1^ NeuroPhysiology & Behavior Laboratory Centro Regional de Investigaciones Biomédicas School of Medicine of Ciudad Real University of Castilla‐La Mancha Ciudad Real Spain; ^2^ Departamento de Ciencias Biomédicas Básicas European University of Madrid Madrid Spain; ^3^ Instituto de Neurociencias de Castilla y León University of Salamanca Salamanca Spain

**Keywords:** amyloid‐β_1–42_ peptide, brain slices, GirK channels, habituation, hippocampus, LABORAS, synaptic plasticity

## Abstract

Hippocampal synaptic plasticity disruption by amyloid‐β (Aβ) peptides + thought to be responsible for learning and memory impairments in Alzheimer's disease (AD) early stage. Failures in neuronal excitability maintenance seems to be an underlying mechanism. G‐protein‐gated inwardly rectifying potassium (GirK) channels control neural excitability by hyperpolarization in response to many G‐protein‐coupled receptors activation. Here, in early in vitro and in vivo amyloidosis mouse models, we study whether GirK channels take part of the hippocampal synaptic plasticity impairments generated by Aβ_1–42_. In vitro electrophysiological recordings from slices showed that Aβ_1–42_ alters synaptic plasticity by switching high‐frequency stimulation (HFS) induced long‐term potentiation (LTP) to long‐term depression (LTD), which led to in vivo hippocampal‐dependent memory deficits. Remarkably, selective pharmacological activation of GirK channels with ML297 rescued both HFS‐induced LTP and habituation memory from Aβ_1–42_ action. Moreover, when GirK channels were specifically blocked by Tertiapin‐Q, their activation with ML297 failed to rescue LTP from the HFS‐dependent LTD induced by Aβ_1–42_. On the other hand, the molecular analysis of the recorded slices by western blot showed that the expression of GIRK1/2 subunits, which form the prototypical GirK channel in the hippocampus, was not significantly regulated by Aβ_1–42_. However, immunohistochemical examination of our in vivo amyloidosis model showed Aβ_1–42_ to down‐regulate hippocampal GIRK1 subunit expression. Together, our results describe an Aβ‐mediated deleterious synaptic mechanism that modifies the induction threshold for hippocampal LTP/LTD and underlies memory alterations observed in amyloidosis models. In this scenario, GirK activation assures memory formation by preventing the transformation of HFS‐induced LTP into LTD.

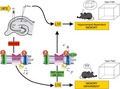

## Graphical abstract

Disruption of hippocampal synaptic plasticity by amyloid‐β (Aβ) peptides is thought to be responsible for learning and memory impairments in early Alzheimer's disease. Here we describe an Aβ‐mediated deleterious synaptic mechanism that modifies the threshold for the induction of hippocampal LTP and/or LTD and underlies memory alterations in amyloidosis models. We also propose a potential intervention to prevent such synaptic impairment through G‐protein‐gated inwardly rectifying potassium (GirK) channels activation.

**Abbreviations used**
aCSF: artificial cerebrospinal fluid
AD: Alzheimer's disease
Aβ: amyloid‐β
fEPSP: field excitatory post‐synaptic potential
GirK: G‐protein‐gated inwardly rectifying potassium channels
HFS: high‐frequency stimulation
*I.c.v.*: intracerebroventricular
LTD: long‐term depression
LTP: long‐term potentiation
NMDAR: NMDA receptor
RRID: Research Resource Identifier
TQ: tertiapin‐Q

## 1 INTRODUCTION

One important mechanism controlling the excess of excitation in neurons relies on G‐protein‐gated inwardly rectifying potassium (Kir3/GirK) channels. There are four primary neuronal GIRK subunits, GIRK1‐4 (Glaaser & Slesinger, 2015), although in the hippocampus prototypical GirK channels are formed by GIRK1⁄GIRK2 heteromultimers (Lujan, Fernandez, Velasco, Aguado, & Wickman, 2014). At resting membrane potential values, GirK activation generates an outward K^+^ current that decreases neuronal excitability by hyperpolarization (Luscher & Slesinger, 2010). They mediate inhibitory responses as effectors of a variety of G‐protein‐coupled receptors for neurotransmitters such as GABA (GABA_B_), 5‐HT (5HT‐1A), adenosine (A1R), dopamine (D2 and D4), opioids (CB1), or somatostatin (Glaaser & Slesinger, 2015). GirK channels *gain‐of‐function* leads to models of Down syndrome or Parkinson's disease, whereas *loss‐of‐function* underlies disorders such as epilepsy, addiction or pain (Luscher & Slesinger, 2010), suggesting their main role as regulators for the fine tuning of excitation/inhibition physiological homeostasis.

Alzheimer's disease (AD) has been associated with high levels of amyloid‐β (Aβ) peptides in the brain (Selkoe & Hardy, 2016). Evidence supports the observation that Aβ induces subtle changes in the balance between excitatory and inhibitory neurotransmission during preclinical states of AD (Palop & Mucke, 2010). Such imbalance leads to neuronal hyperactivity that constitutes an early neuronal dysfunction (Busche & Konnerth, 2015) which has consequences at the synaptic, circuits and neural networks, and behavioral levels (Busche & Konnerth, 2016; Goutagny & Krantic, 2013; Palop & Mucke, 2010; Zott, Busche, Sperling, & Konnerth, 2018). Hippocampal circuits dysfunction because of Aβ‐induced hyperactivity appears as one of the initial symptoms in the pathogenic cascade of AD (Busche & Konnerth, 2016). Neural hyperactivity is accompanied by impairments of hippocampal oscillatory activity and loss of synaptic plasticity mechanisms (Kurudenkandy et al., 2014; Palop & Mucke, 2016; Sanchez‐Rodriguez, Gruart, Delgado‐Garcia, Jimenez‐Diaz, & Navarro‐Lopez, 2019; Sanchez‐Rodriguez et al., 2017; Varga et al., 2015), causing alterations of hippocampal long‐term synaptic potentiation (LTP) and depression (LTD) (Styr & Slutsky, 2018), the cellular substrates of memory (Lomo, 2018; Luscher & Malenka, 2012). All these factors underlie the deterioration of hippocampal cognitive functions. Although most of the mechanisms for Aβ‐impairing excitability are still unknown, therapies focused on neuronal inhibition enhancement are showing very promising results in animals, and in humans (Bakker et al., 2012; Vossel, Tartaglia, Nygaard, Zeman, & Miller, 2017). Thus, GirK channels emerge as an interesting tool based on their inhibitory role as key regulators of neuronal excitability in the hippocampus (Slesinger, 2015).

It has been previously reported that Aβ induces a reduction in gene expression of hippocampal GirK subunits (Mayordomo‐Cava, Yajeya, Navarro‐Lopez, & Jimenez‐Diaz, 2015) and decreases GirK conductance inducing an increase in excitability in CA3 pyramidal neurons (Nava‐Mesa, Jimenez‐Diaz, Yajeya, & Navarro‐Lopez, 2013). We also found in behaving mice that GirK‐dependent signal enhancement rescued hippocampal functions in an in vivo mouse model of early Aβ pathology (Sanchez‐Rodriguez et al., 2019, 2017). Here, we focused on the mechanisms by which hippocampal synaptic plasticity is altered in amyloidopathy models (Styr & Slutsky, 2018). It is well stablished that high‐frequency stimulation (HFS) applied to the *Schaffer* collaterals induces LTP of the synaptic responses (Bliss, Collingridge, Morris, & Reymann, 2018) but in the present work we show that Aβ transforms HFS‐induced LTP into LTD with subsequent memory formation impairment. Interestingly, this Aβ‐deleterious action can be avoided by selective enhancement of GirK—dependent signaling supporting the hypothesis of counteracting neuronal hyperactivity as a potential tool to restore excitability levels and upstreaming functions lost in AD brain (Busche & Konnerth, 2015; Nava‐Mesa, Jimenez‐Diaz, Yajeya, & Navarro‐Lopez, 2014; Palop & Mucke, 2016; Styr & Slutsky, 2018).

## 2 MATERIALS AND METHODS

### 2.1 Animals

**Figure 1:**
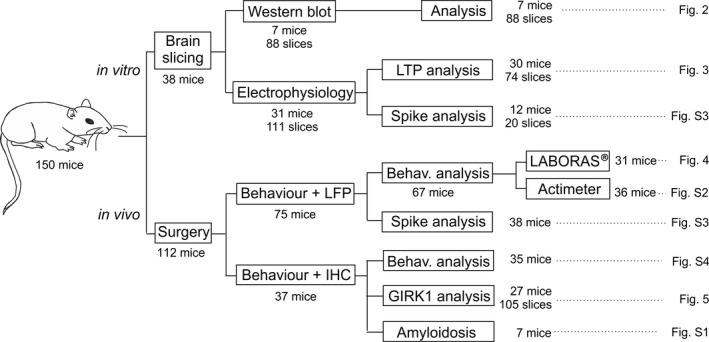
Scheme of animals, experiments and corresponding figures. IHC, immunohistochemistry; LTP, long‐term potentiation; LFP, local field potential; LABORAS^®^, Laboratory Animal Behavior, Observation, Registration, and Analysis System

Experiments detailed in the flowchart (Figure 1) were carried out on 150 C57BL/6 male mice (RRID:MGI:5,656,552) aged 3–10 weeks old (10–25 gr) obtained from an authorized distributor (Charles River). Mice were housed in group cages (*n* = 5) before surgeries, after which they were placed in individual cages and a piece of environmental enrichment for rodents was provided. They had ad libitum access to food and water throughout all experiments. All animal procedures were reviewed and approved by the Ethical Committee for Use of Laboratory Animals of the University of Castilla‐La Mancha (Ref: PR‐2018‐05‐11), and followed the European Union guidelines (2010/63/EU) and Spanish regulations for the use of laboratory animals in chronic experiments (RD 53/2013 on the care of experimental animals: BOE 08/02/2013). The study was not pre‐registered and was exploratory.

### 2.2 Hippocampal slice preparation for electrophysiology

Hippocampal slices were prepared as described previously (Nava‐Mesa et al., 2013). Briefly, animals were deeply anesthetized with halothane (Fluothane; AstraZeneca) as suggested by the Ethical Committee because of its high efficacy and quick action and received buprenorphine intramuscularly as analgesic (0.01 mg/kg; # 062,009, BUPRENODALE®; Albet). They were intracardically perfused with 1 ml oxygenated (95% O_2_ + 5% CO_2_) ice‐cold (4–6°) artificial cerebrospinal fluid (aCSF), modified with sucrose (234 mM, #84100; Sigma) replacing the NaCl (118 mM, #S9888; Sigma) to minimize damage, and decapitated. The brain was excised and rapidly immersed in oxygenated ice‐cold modified aCSF. Horizontal brain slices (300‐µm thick) were obtained with a vibratome (7000smz‐2; Campden Instruments). Slices were incubated in oxygenated normal aCSF (containing in mmol/L: 118 NaCl, 3 KCL (#P3911; Sigma), 1.5 CaCl_2_ (#499609; Sigma), 1 MgCl_2_ (#208337; Sigma), 25 NaHCO_3_ (#S6014; Sigma), 30 Glucose (#G8270; Sigma) and 1 NaH_2_PO_4_ (#S8282; Sigma)_,_ pH = 7.35) 1 hr at 30°C and then, for at least 1 hr, at room temperature (22°C) before the recordings.

For electrophysiological recordings a single hippocampal slice (3–4 per animal, *n* = 23) was transferred to an interface recording chamber (BSC‐HT and BSC‐BU; Harvard Apparatus) and perfused continuously with aCSF. Extracellular field potentials from de CA1 pyramidal neurons were recorded using a borosilicate glass micropipette (1–3 MΩ; RRID:SCR_008593; World Precision Instruments) filled with aCSF positioned on the slice surface in the *stratum radiatum* of CA1 region of the hippocampus and connected to the headstage of an extracellular recording amplifier (NeuroLog System; Digitimer). The synaptic responses were evoked by single pulses stimulation applied at 0.5 Hz on the *Schaffer* collaterals pathway through a tungsten concentric bipolar stimulating electrode (TM33CCINS‐B; World Precision Instruments) using a programmable stimulator (MASTER‐9; A.M.P.I). Biphasic, square‐wave pulses of 1 ms duration were adjusted to the intensity necessary for evoking ≈40% of a maximum field excitatory post‐synaptic potential (fEPSP) response.

For long‐term potentiation (LTP) induction, a high‐frequency stimulation (HFS) was used consisting of 100 Hz trains of 1‐s duration repeated five times with a 30‐s inter‐train interval (Schuette, Fernandez‐Fernandez, Lamla, Rosenbrock, & Hobson, 2016). The stimulus intensity was also set to ≈40% of its asymptotic values. Baseline values for the amplitude of fEPSPs evoked at the CA3—CA1 synapse were collected at least 10 min prior to LTP induction. After LTP induction fEPSPs were recorded during at least 30 min.

### 2.3 Western blotting

Hippocampal slices (3–4 per condition and per animal, *n* = 7) were incubated at room temperature in two different conditions, (a) ACSF or (b) ACSF enriched with Aβ_1–42_ (0.5 μM), during different time periods (0, 30, 120 min). Time points for protein expression analysis where chosen based on our previous in vitro results (Mayordomo‐Cava et al., 2015). GIRK1 and 2 were evaluated as their heterotetramers are the prototypical Girk channels in the hippocampus (Fernandez‐Alacid, Watanabe, Molnar, Wickman, & Lujan, 2011). Finally, the slices were collected after incubation and frozen at −80°C at each time point until further processing. Proteins were extracted in 150 μl RIPA buffer (#R0278; Sigma) by blender homogenization, incubated for 2 hr on ice and centrifuged at 12,000 g to remove the debris. The proteins were diluted to yield 1 mg/ml and stored in aliquots at −80°C. 10 μg of total protein were used for the electrophoresis with the Mini Protean 3 Cell System (Bio‐Rad) and resolved in 10% acrylamide gels as described elsewhere (Vega‐Avelaira, Moss, & Fitzgerald, 2007). Following the blot, membranes were blocked in PBS solution (#P4417; Sigma) with 0.1% Tween 20 (#P9416; Sigma) and 4% semi‐skimmed milk powder (Nestle). The primary antibodies were incubated at 4°C overnight with gentle shaking and diluted in blocking buffer (rabbit anti‐GIRK1 [#APC‐005] or rabbit anti‐GIRK2 [#APC‐006] at 1:500 from Alomone or mouse anti‐beta‐tubulin at 1/2000 [#T8328; Sigma]). Secondary antibodies (anti‐rabbit IgG‐HRP [#AP307] and anti‐mouse IgG‐HRP [#AP130P]) from Sigma were used at a dilution of 1/2000. Antibody excess was removed with six washes in PBS/0.1% Tween 20. To reveal the signal, we used the ECL western blotting detection system from Amersham biosciences (Buckinghamshire) and the images were acquired with the ChemiDoc™ MP Imaging System (Bio‐Rad). The intensity of the western blots bands for anti‐GIRK1 (~60 kDa), anti‐GIRK2 (~45 Kda) were measured by densitometry with Image Lab 6.0.1 Software (Bio‐Rad) and beta‐tubulin (~50 kDa) as housekeeping gene for normalization. Values were expressed as the ratio in percentage “densitometry protein/densitometry β‐tubulin” named as “mean intensity normalized” and represented with the standard deviation.

### 2.4 Surgery for intracerebroventricular drug injections in alert animals

For the intracerebroventricular (*i.c.v.*) administration of drugs included in this study, C57BL/6 (*n* = 68, 24–34 g) male mice were prepared as previously described (Sanchez‐Rodriguez et al., 2019, 2017). Mice were anesthetized with ketamine/xylazine administered intraperitoneally (75/10 mg/Kg; KETALAR®; Pfizer and ROMPUM®; Bayer), following the guidelines and authorization of the Ethical Committee of the University of Castilla‐la Mancha because of their deep, stable and prolonged anesthetic effect; and implanted chronically with a blunted, stainless steel, 26‐G guide cannula (Plastic One) in the ventricle (0.5 mm posterior to bregma, 1.0 mm lateral to midline, and 1.8 mm below the brain surface (Paxinos & Franklin, 2001), fixed to the skull with the help of two small screws and dental cement (Figure 4a). Buprenorphine was administered intramuscularly as analgesic during and after surgery (0.01 mg/kg; # 062,009, BUPRENODALE®; Albet). Injections were carried out with a 33‐G internal cannula, 0.5 mm longer than the implanted guide cannula and inserted inside it. Mice were allowed a week for recovery before experimental sessions. After surgery a healing cream (Blastoestimulina; Almirall) was applied to accelerate recovery and decrease animal suffering. Handling was performed routinely to minimize stress to the mice during experimental manipulation. Injections were performed in freely moving mice with the help of a motorized Hamilton syringe at a rate of 0.5 μl/min. In order to avoid bias and blind the experimenter to treatment, experimental group assignment (both drug code and animal) in each experiment was performed by a different person than the experimenter. Drugs were coded by numbering each drug from #*X* = 1 to 5 in all experiments except in Figure 4 were the number of drugs was #*X* = 3). Likewise, each animal was coded by numbering them arbitrarily from 1 to #*Y* (#*Y* = total number of animals in the experiment). No randomization was performed to assign numbers to drugs or animals in each experiment. Then, animals and drugs were matched for treatment following the order of the coding numbers in repeated sequences. Thus, animals were treated and assessed following the repeated sequences (an example would be as follows: sequence of drugs # 1,2,3: drug #1 to animal #1, drug #2 to animal #2, drug #3 to animal #3; code sequence for drugs starts again: drug #1 to animal #4, drug #2 to animal #5, etc.). Animals were assigned to each experimental group in each experiment after assessing that they were fully recovered and did not present any signs of pain, suffering or altered behavior after surgeries. Three animals were excluded from further experiments after surgery as they showed ≥25% weight loss. These animals were not replaced, resulting in accordingly lower final numbers of animals for the respective experiments.

Animals prepared in the way described above (*i.e*., chronic guide cannula implanted in the left ventricle) were used for behavioral testing and immunohistochemical experiments. As described in Figure 1, one cohort of animals was used for open field habituation testing in the LABORAS® system (see corresponding sections below). A second cohort of mice was used for rota‐rod testing (see Supporting Information, Figure S4) and immunohistochemistry to study GIRK1 expression in the dorsal hippocampus (details below). In addition, some animals *i.c.v.*‐injected with Aβ_1–42_ (or vehicle) were also used to study the peptide diffusion in the brain. For that purpose, immunohistochemistry against Aβ_1–42_ was performed (Supporting Information, Figure S1).

An additional cohort of animals was prepared for the chronic recording of local field potentials from the CA1 region of the hippocampus together with chronic implantation of a guide cannula in the ventricle. Animals operated this way were used for both open field habituation memory testing in an actimeter (Supporting Information, Figure S2) and to asses excitability of the hippocampus in our *i.c.v.‐*injected experimental groups (Supporting Information, Figure S3).

### 2.5 Open field habituation test

Habituation is an elementary form of non‐associative hippocampal‐dependent learning. In rodents, habituation to a novel environment is defined as a change in exploratory or locomotor activity with repeated exposures (Leussis & Bolivar, 2006). In this study, re‐exposure to an open field (habituation test) was used to test this form of learning. Animals performed one trial per day in two consecutive days. Trial 1 on day 1 was the initial exposure to the open field (OF1 or training trial), and trial 2 was a 24 hr later re‐exposure to the arena (OF2, retention or habituation trial). *I.c.v.* injections were performed 1 hr before retention trial. Either saline, soluble Aβ_1–42_, or Aβ_1–42_ + ML297 were injected through a guide cannula as described above. On each trial, mice were placed in the center of the open field arena and allowed to explore for 15 min. Animal movements were automatically recorded for the 15‐min period using a LABORAS® system (*Laboratory Animal Behavior, Observation, Registration, and Analysis System*, Metris). The open field was a LABORAS® cage made of 40 × 23 × 4 cm Plexiglas base arena and a 40 × 23 × 11 cm top. Total traveled distance was assessed by LABORAS® system based on detecting vibrations of the movements of each mouse. All data were digitized and analyzed using Metris software. The arena was cleaned with 70% EtOH and allowed to dry between animals to remove odors. Injections and behavioral tests were performed in the morning timeframe (9 a.m. to 2 p.m.).

### 2.6 Histology, immunochemistry, and image analysis

After behavioral experiments were performed, some animals from each experimental group (*n* = 7 animals per group) were selected for histological and immunohistochemical studies. Mice were deeply anesthetized with ketamine/xylazine administered intraperitoneally (75/10 mg/Kg; KETALAR®; Pfizer and ROMPUM®; Bayer), following the guidelines of the Ethical Committee of the University of Castilla‐la Mancha because of their deep and prolonged anesthetic effect. They received buprenorphine intramuscularly as analgesic (0.01 mg/kg; # 062,009, BUPRENODALE^®^; Albet), and were perfused transcardially with 0.9% saline (#S9888; Sigma) followed by 4% paraformaldehyde (#141451; Panreac Applichem) prepared in phosphate‐buffered saline (PBS; #P4417; Sigma; 0.1 M, pH 7.4). Their brains were removed and cryoprotected in 30% sucrose (#84100; Sigma) in PB. Coronal sections (40 µm) were obtained using a sliding freezing microtome (Microm HM 450) and collected serially in a solution of glycerol (#G7757; Sigma)‐PBS (1:1) for storage at −20°C. Selected sections including the implanted cannula sites were mounted on gelatinized glass slides and stained using the Nissl technique with 0.25% Thionine (#8893; Sigma) to determine the location of the implanted cannula.

For fluorescence immunohistochemistry, free‐floating sections were blocked with 10% normal donkey serum (RRID:AB_2810235; Sigma) in Tris‐buffered saline (TBS) containing 0.1% Triton X‐100 (#T8532; Sigma; TBS‐T) for 45 min, and then incubated overnight at room temperature with polyclonal rabbit anti‐GIRK1 (1:400; RRID:AB_2571710, Frontier Institute) primary antibody in TBS‐T with 0.05% sodium azide (#S/2360/48; Fisher Scientific) and 5% NDS. The following day, sections were rinsed in TBS‐T (3 × 10 min) and incubated for 2 hr at room temperature with 1:150 dilutions of FITC‐conjugated donkey anti‐rabbit (RRID:AB_2315776; Jackson Immuno Research) in TBS‐T. After several washes with TBS (3 × 10 min), slices were incubated in 0.01% DAPI (#sc‐3598; Santa Cruz Biotechnology) in TBS for 5 min. Finally, sections were washed with TBS (3 × 10 min), mounted on gelatinized glass slides, dehydrated and coverslipped using a fluorescence mounting medium (#S3023; Dako mounting medium, Agilent). Images were acquired by confocal microscopy using a laser scanning microscope (LSM 800; Carl Zeiss). GIRK1 subunit expression was calculated from 3 × 4‐stitched images, at 10× magnification, using ImageJ software (RRID:SCR_003070; NIH). Randomly selected squares of approximately 15 × 15 μm through the *stratum lacunosum‐moleculare* were used to measure the intensity of GIRK1 inmunostaining (*i.e*. optical density) at the dorsal hippocampus. GirK channels are mainly expressed in the dendrites of pyramidal neurons in the hippocampus and these expression is related to their role in controlling glutamatergic inputs, especially those coming from the entorhinal cortex through the perforant pathway, which are distributed along the *stratum lacunosum‐moleculare* (Lujan & Aguado, 2015). Therefore, immunolabeling for GIRK1 is intense in this CA3‐CA1 dendritic layer. Thus, we hypothesized that possible optic density variations between experimental groups could be measured more accurately in this area, because of the greater range of measurement values. Mean background level, obtained from four different squares from non‐stained areas, was subtracted, and the values of optical density were normalized with respect to the control (vehicle) group values. Data were expressed as mean ± *SEM*.

### 2.7 Chemicals and their application

All drugs were stored at −20°C as concentrated aliquots in distilled water and applied by superfusion to the slice at a rate of 3 ml/min. ML297 (#ab143564; Abcam) was dissolved in PBS with the help of a shaker and/or sonicator, aliquoted (0.5 mM) and perfused at 10 μM (Kotecki et al., 2015), whereas tertiapin‐Q (TQ) (#T1567; Sigma) was perfused at 0.5 μM (Nava‐Mesa et al., 2013). Stock solutions of synthetic human Aβ_1–42_ and its reverse control Aβ_42‐1_ (#4014447 and #4027991, respectively, both from Bachem) were prepared in 0.1% NH_4_OH (#338818; Sigma) at a concentration of 0.2 mM (Teplow, 2006; Ulrich, 2015) and perfused at 0.5 µM (Tamagnini, Scullion, Brown, & Randall, 2015). For western blotting hippocampal slices were incubated at same concentration. To model focal Aβ pathology in the hippocampus in vivo, we selected a non‐transgenic mouse model we have previously used (Sanchez‐Rodriguez et al., 2019, 2017), that resembles initial preclinical stages of the disease and enables evaluating the key role of early amyloid forms in AD. Briefly, Aβ_1–42_ was dissolved in vehicle and incubated 1 hr at room temperature before injection to form highly toxic prefibrillar oligomers (Jan, Hartley, & Lashuel, 2010). For *i.c.v.* injection, 3 μg of Aβ_1–42_ was dissolved in 3 μl of vehicle and injected through the guide cannula using a Hamilton syringe at a rate of 0.5 μl/min. Drug diffusion after *i.c.v.* injection was studied confirming that was mainly restricted to dorsal hippocampal formation (Supporting Information Figure S1). For the Aβ_1–42_ + ML297 group, sequential 3 µl‐injections of each drug were made at 15 min intervals (Aβ_1–42_ administration took place first, and ML297 (1.5 mM) was injected 15 min later) to both assure small volume injections at a time and avoid any flow back when removing the internal cannula.

### 2.8 Data analysis

**Figure 2:**
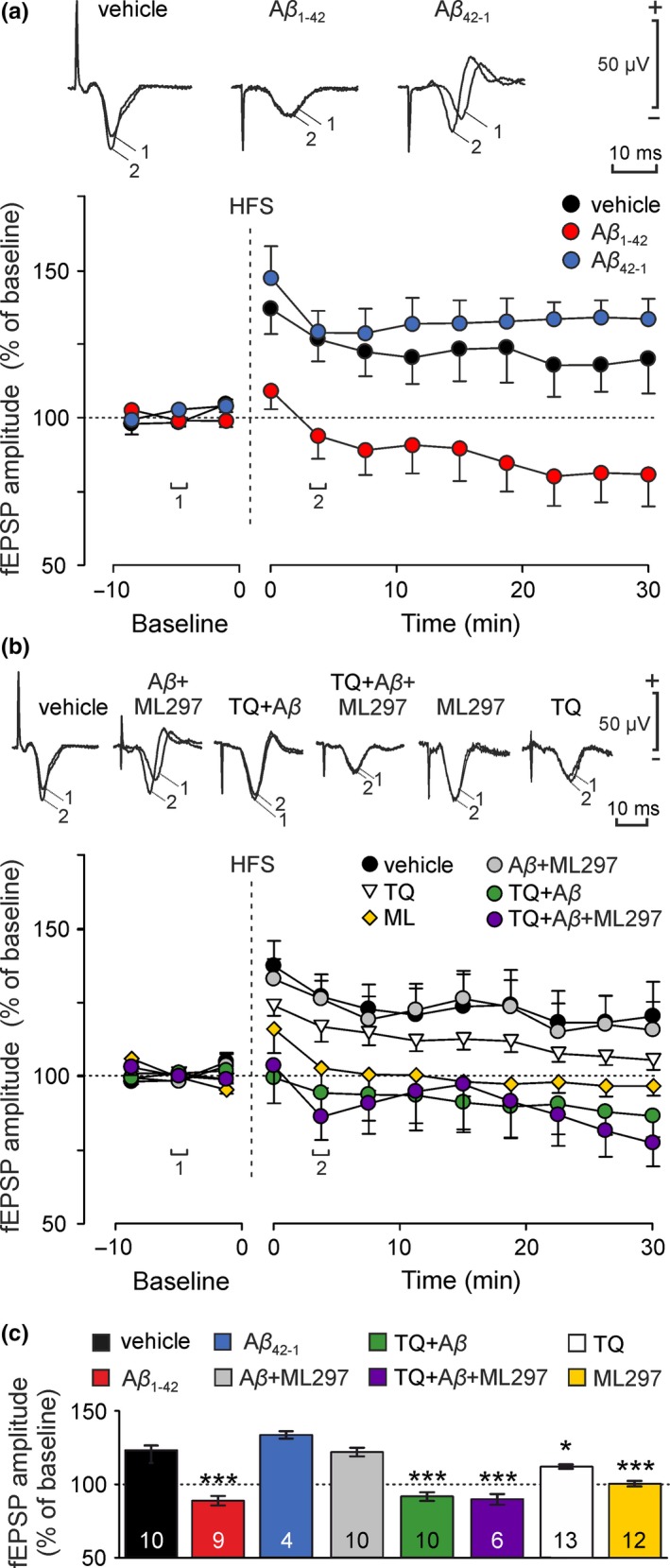
Effect of amyloid‐β (Aβ)_1–42_ and G‐protein‐gated inwardly rectifying potassium channels (GirK) signaling modulation on long‐term potentiation (LTP) in vitro. Hippocampal slices were perfused with Aβ_1–42_ (0.5 μM), Aβ_42‐1_ (0.5 μM), ML297 (10 μM), TQ (0.5 μM) or different combinations of Aβ_1–42_, ML297 and TQ. A baseline was stablished by stimulating *Schaffer* collaterals at 0.5 Hz. LTP was induced by an high‐frequency stimulation (HFS) protocol, and evolution of field excitatory post‐synaptic potential (fEPSP) amplitude was analyzed during the following 30 min. Representative examples of averaged (*n* = 20) fEPSPs at time (1) Baseline; and (2) after HFS, are illustrated in (a) and (b) for each experimental group. (a) Data represent fEPSP amplitude after LTP induction in control (vehicle), Aβ_1–42_ and Aβ_42‐1_‐treated slices. There were significant differences in potentiation levels between experimental groups (*F*_(2,20)_ = 6.23; *p* = .008). Aβ_1–42_ prevented LTP and induced long‐term depression (LTD) (*post hoc* vs.* *control, *p* = .015). In Aβ_42‐1_‐treated slices, LTP did not differ from the control vehicle group (*post hoc *vs. control, *p* = .726). (b) Plot representing fEPSP amplitude after LTP induction in control (vehicle), TQ, ML297, Aβ_1–42_ + ML297, TQ + Aβ_1–42_, and TQ + Aβ_1–42_ + ML297‐treated slices. Potentiation levels between experimental groups were significantly different (*F*_(5,55)_ = 4.77; *p* = .001). Note that after decreasing GirK‐dependent signal with TQ, the LTP induced was lower than control's (*post hoc* TQ vs. control, *p* = .042), whereas GirK channel activation with ML297 hindered LTP (*post hoc* ML297 vs. control, *p* = .03). In contrast, an increase in GirK channels conductance restored the LTP abolished by Aβ_1–42_ (*post hoc* Aβ_1–42_ + ML297 vs. control, *p* = .998). However, Aβ_1–42_ prevents LTP (inducing LTD) even when GirK channels were previously closed by TQ (*post hoc* TQ + Aβ_1–42_ vs. control, *p* = .005). Blocking GirK channels also prevented restoration of LTP by ML297 in our in vitro model of amyloidopathy (*post hoc* TQ + Aβ_1–42_ + ML297 vs. control vehicle, *p* = .012). (c) For each experimental group, bars illustrate potentiation level in the 30 min following HFS protocol as the mean of all measured post‐HFS points (9 potentiation measures per treatment/slice in 30 min) from every slice (4–13 slices per treatment). Number of slices for each condition is indicated in the corresponding bar. n numbers for each condition = number of total independent potentiation points = number of slices for each condition multiplied by nine potentiation measures per slice. Error bars represent the standard error of the mean. *p < .05; ***p < .001

**Figure 3:**
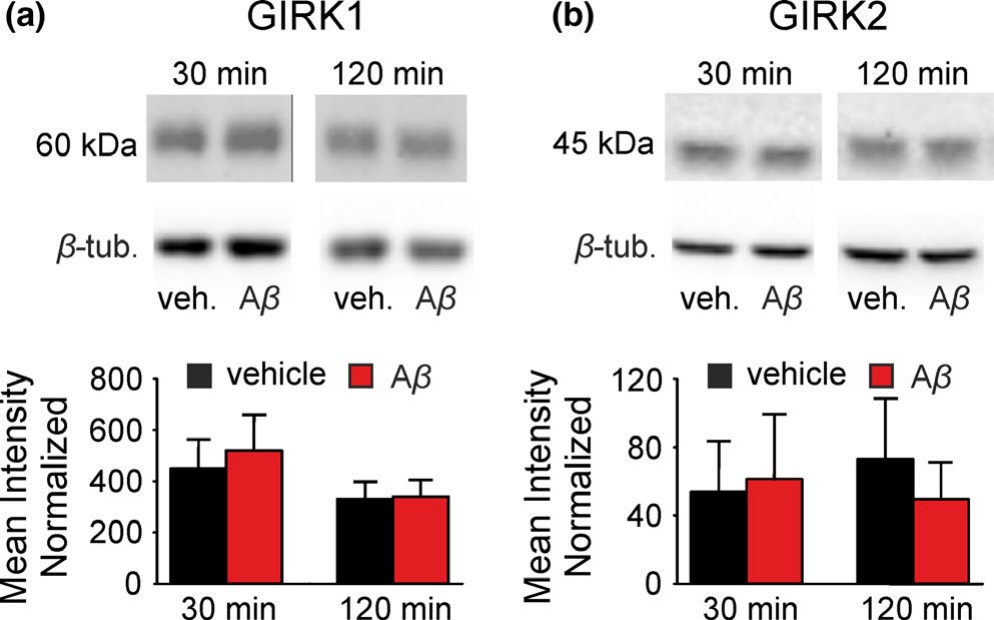
Effects of Aβ_1–42_ on hippocampal G‐protein‐gated inwardly rectifying potassium channels (GirK) protein expression pattern in vitro. Western‐blot analysis of the (a) GIRK1 and (b) GIRK2 protein levels in hippocampus slices treated with Aβ_1–42_ (0.5 μM) or vehicle (control group: vehicle, veh.) for 30 and 120 min. Results are expressed with the standard deviation; (*n* = 21–28 slices per experimental group, from seven mice). Aβ, amyloid‐β; β‐tub, β‐tubulin

**Figure 4:**
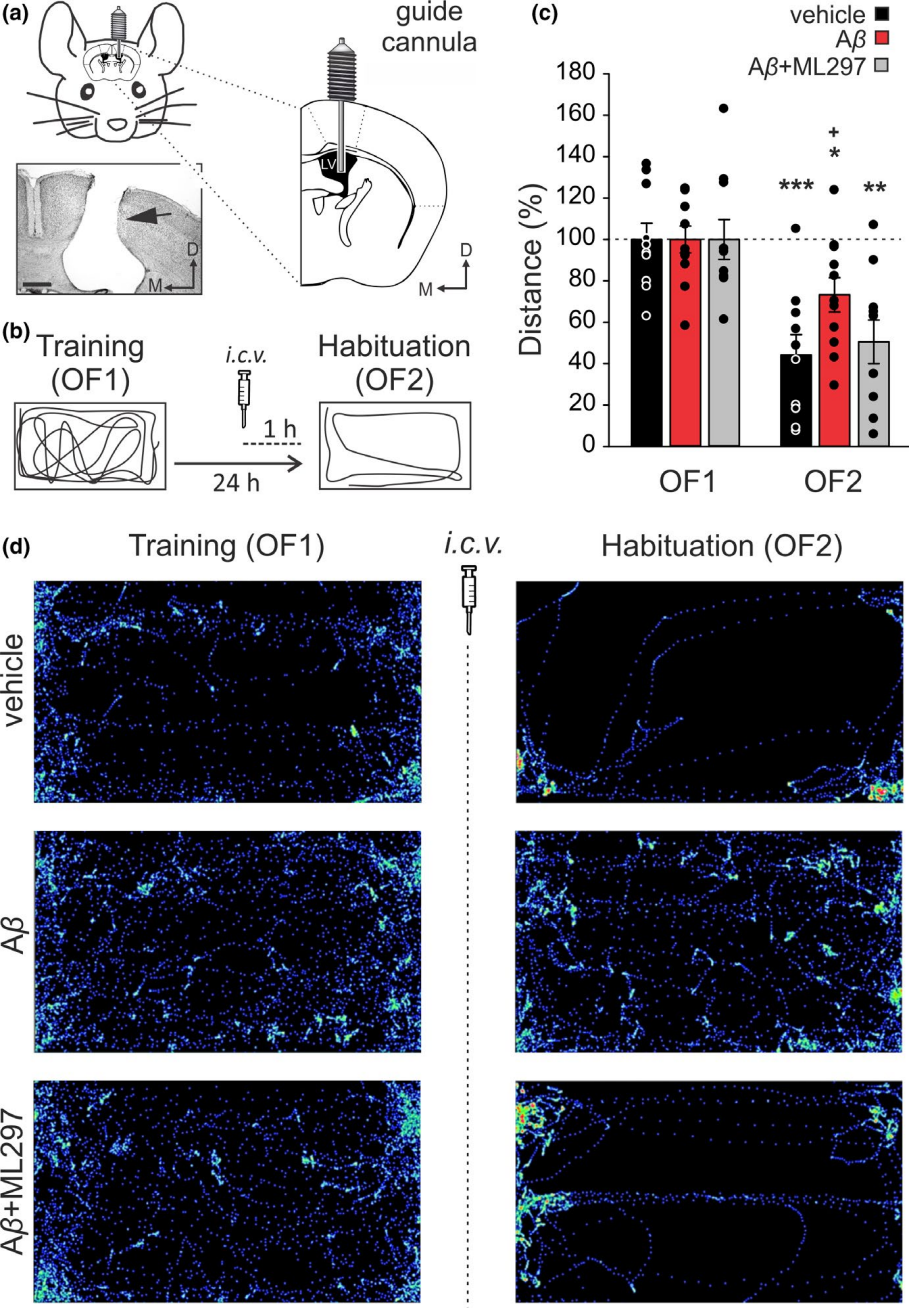
Effects of G‐protein‐gated inwardly rectifying potassium channels (GirK) activation on mice intracerebroventricular (*i.c.v.*)‐injected with amyloid‐β (Aβ)_1–42_ during hippocampal‐dependent habituation to an open field. (a, b) Experimental design. (a) Pictures illustrate how mice were prepared for drug administration. A stainless‐steel guide cannula was implanted chronically on the left ventricle [1 mm lateral, 0.5 mm posterior to bregma, and 1.8 mm from the brain surface]. The photomicrograph serves as histological verification of cannula position (black arrow). Scale Bar 500 µm. LV, Lateral Ventricle; D, dorsal; M, medial. (b) Open field habituation test. Mice were exposed for 15 min to the same open field (OF) two consecutive times with a 24‐hr interval (OF1, Training trial, and OF2, Habituation or retention trial). Habituation memory levels were determined by measuring exploration behavior. *I.c.v*. injections were performed 1 hr before OF2. For each mouse, traveled distance was automatically tracked and recorded using a LABORAS® system (Metris, Hoofddorp, The Netherlands) on the basis of detecting vibrations of the movements of each animal. Diagrams represent an example of the path followed by a control (vehicle‐injected) animal during both training (OF1) and habituation (OF2) sessions. (c) Total horizontal distance traveled during the 15‐minsession for vehicle (*n* = 10 animals), Aβ_1–42_ (*n* = 11) and Aβ_1–42_ + ML297 (*n* = 10) ‐treated mice before (OF1) and after (OF2) drug administration. Data were normalized as percentage of the movement in the training (OF1) session. Differences between OF1 and OF2 are indicated by asterisks (*p < .05; **p < .01; ***p < .001). Differences versus*.* control (vehicle) within OF2 session are indicated by crosses (+, *p *< .05). (d) LABORAS®‐generated images illustrate the distance and path traveled by a representative animal of each experimental group during OF1 and OF2 sessions

**Figure 5:**
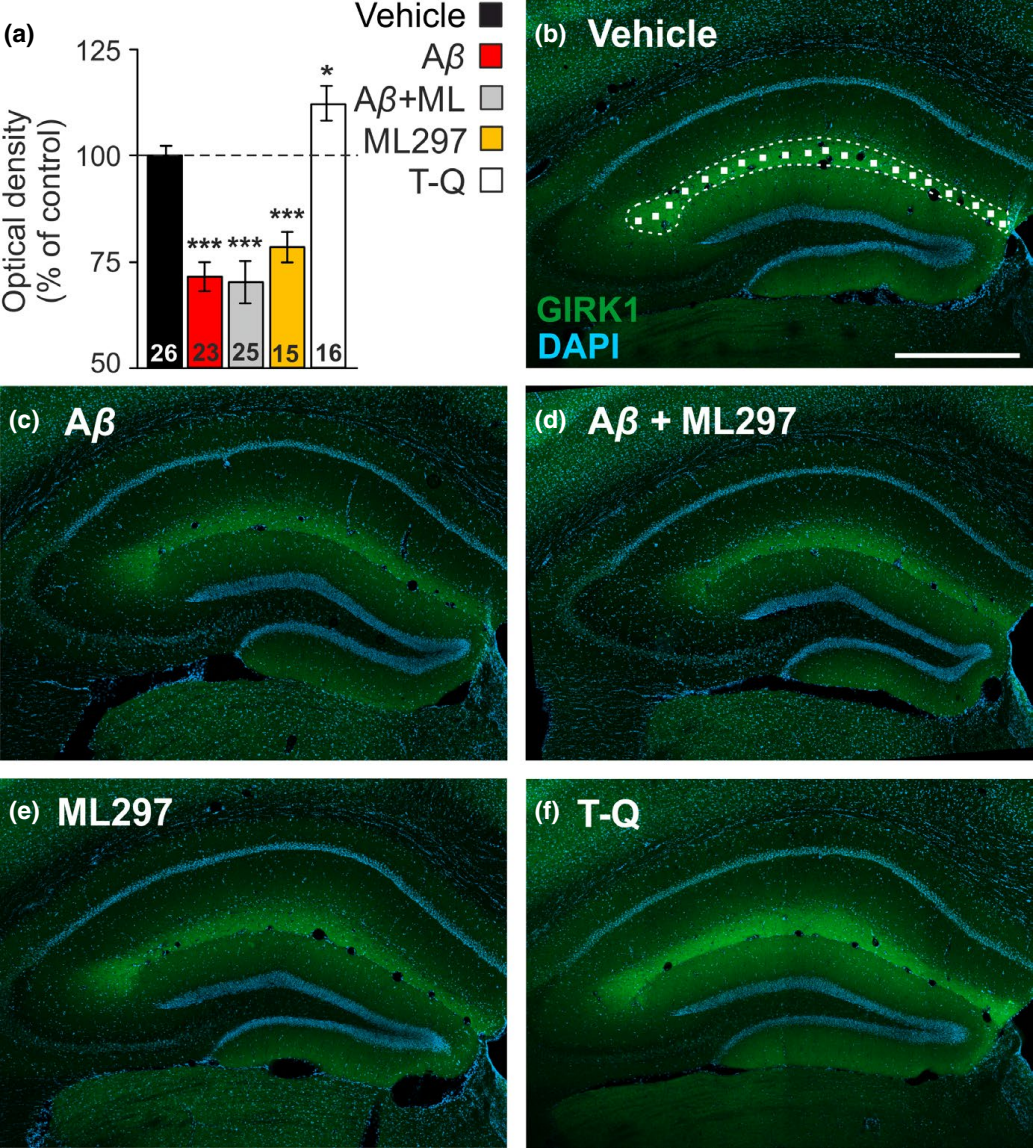
Hippocampal G‐protein‐gated inwardly rectifying potassium channels (GirK) protein pattern is altered by amyloid‐β (Aβ)_1–42_ in vivo. (a) Bar plots showing GIRK1 immunostaining intensity (measured as optical density), as percentage of control (vehicle) values (dashed line, 100%) in the *stratum lacunosum‐moleculare* of the dorsal hippocampus in intracerebroventricular (*i.c.v.*)‐injected mice with vehicle (*n* = 7 animals), Aβ_1–42_ (*n* = 6), Aβ_1–42_ + ML297 (*n* = 6), ML297 (*n* = 4) or tertiapin‐Q (TQ) (*n* = 4). Number of hippocampal sections for each condition (*n* = 15–26 slices) is indicated in the corresponding bar. Differences versus*.* control (vehicle) are indicated by asterisks (*p < .05; ***p < .001). (b) Confocal fluorescence photomicrograph showing the distribution of GIRK1 subunit (green labeling), with intense immunolabeling in the *stratum lacunosum‐moleculare*, and DAPI stained cells (blue labeling) in the dorsal hippocampus of a representative vehicle‐injected mouse. The image illustrates how random 15 × 15 µm squares were distributed through the *stratum lacunosum‐moleculare* to measure GIRK1 optical density. Calibration bar: 500 µm (c–f) Representative confocal microscopy images showing GIRK1 immunostaining in *i.c.v.*‐injected mice with Aβ_1–42_ (c), Aβ_1–42_ + ML297 (d), ML297 (e), and TQ (f). Calibration bar in (b) also applies for (c–f)

Blinding was achieved in all experiments as experimenters were always unaware of the animal's treatment during in vitro or behavioral experimentation and while performing all different analysis. Statistical analysis of collected data was performed with SPSS 15.0 (RRID:SCR_002865). Normal distribution of the data was tested with Kolmorov–Smirnov's test. Homogeneity of variances was assessed with Levene's test. When the distribution of the variables was normal Student's *t *test (Figure 3, western blot data; Figures S2 and S3 in vivo data), one‐way (Figure 2 in vitro* data;* Figures 3, 4, Figures S2 and S3 *in vivo* data; Figure 5, immunohistochemistry data) or two‐way repeated measures ANOVA (Figure 2 and Figure S4 where *treatment* was the inter‐subject variable, and *time* (Figure 2) or *trial* (Figure S4) the intra‐subject variable) followed by *post hoc* test analysis (DMS or Dunett's methods for multiple comparisons) were performed as needed. When data were not normally distributed the appropriate non‐parametric test was used (Kruskal–Wallis and Mann–Whitney U tests for Figure S2, behavioral data). Statistical significance was determined at a level of *p* ≤ .05.

In vitro recordings were acquired online with the help of a CED 1,401 interface (CED), and stored on a personal computer (sample frequency 12.5 kHz). Data were analyzed with Signal 7 (RRID:SCR_017081) program. As synaptic responses were not contaminated by population spikes, the amplitude (*i.e.*, the peak‐to‐peak value in mV during the rise‐time period) of successively evoked fEPSPs was computed and stored for later analysis. Unless otherwise indicated, the electrophysiological in vitro data are always expressed as mean ± standard error of the mean, and *n* represents the number of averaged slices. Synaptic potentials were averaged (≥5) before quantitative analysis.

Computed results were processed for graphical purposes using the SigmaPlot 12.3 software (RRID:SCR_003210). Final figures were prepared using CorelDRAW v.18 Graphics Suite software (RRID:SCR_014235). Box‐plot was used to rule out outlier values in our data (three animals were excluded from the analysis of the open field habituation test in Figure 4, and 17 slices from the in vitro LTP experiments (Figure 1) because of an abnormal potentiation/depression or the lack of sufficient stability in the recording). No sample size calculation was performed. No exclusion criteria were pre‐determined.

## 3 RESULTS

### 3.1 **Aβ_1–42_ converts HFS‐dependent LTP into LTD at hippocampal CA3**—**CA1 synapse**

LTP has been shown to have a capital role in the hippocampal CA3—CA1 synapse for learning and new memories formation processes (Kumar, 2011). Synaptic plasticity deficits have been previously reported for AD acute models (Eslami, Sadeghi, & Goshadrou, 2018; Kimura, MacTavish, Yang, Westaway, & Jhamandas, 2012). However, the effect of GirK channels modulation on Aβ‐induced LTP impairments has not been deeply investigated. For that purpose, first, HFS was applied at *Schaffer* collaterals to induce LTP in CA1 region in vitro*.* Then the evolution of fEPSPs amplitude evoked in CA1 were compared to baseline for 30 min. This protocol induced a LTP of 123.2 ± 3.2% of baseline in control slices (*n* = 10 slices; black circles, Figure 2a and black bar, Figure 2c. Note that for each experimental group, bars in Figure 2c represent potentiation level in the 30 min after HFS protocol as the mean of the nine points measured post‐HFS from every slice) which remained stable for, at least, half an hour after HFS (*F*_(11,99)_ = 3.86, *p* < .001). However, application of soluble Aβ_1–42_ (0.5 µM) significantly depressed LTP induced by the potentiation protocol to LTD (*n* = 9 slices, Figure 2a, red circles; Figure 2c, 88.7 ± 3.1% of baseline; *post hoc *vs. control, *p* = .015). In contrast, equimolar concentrations of the reverse peptide, Aβ_42‐1_ (0.5 µM), did not significantly affect to LTP (*n* = 4 slices, Figure 2a, blue circles; Figure 2c, 133.6 ± 2.5%; *post hoc *vs. control, *p *= .726), suggesting that LTP inhibition induced by Aβ_1–42_ is therefore, specific.

### 3.2 Aβ_1–42_‐induced depression of hippocampal CA3—CA1 LTP is prevented by GirK‐dependent signal enhancement

GirK‐dependent signal plays an important role as resting conductance for controlling neuronal excess of excitability in CA1 neurons (Drake, Bausch, Milner, & Chavkin, 1997; Kim & Johnston, 2015) and gating hippocampal synaptic plasticity processes (Malik & Johnston, 2017). As neuronal hyperexcitability induces early synaptic dysfunctions in the pathogenesis of AD (Palop & Mucke, 2010) we evaluated in vitro whether increasing GirK conductance impedes the Aβ‐mediated transformation of HFS‐induced LTP into LTD. Slices were sequentially perfused by Aβ_1–42_ (0.5 μM) for 10 min, followed by 10 min with ML297 (10 μM), and then 10 min of baseline before the HFS application. This protocol did assure the induction of the HFS‐dependent LTD before activating GirK channels (Figure 2b). As shown in Figure 2b,c, LTP was induced in the slices treated with Aβ_1–42_ + ML297 (*n* = 10 slices, gray circles and bar; 121.6 ± 2.8% of baseline) without differences with control (vehicle) values (*p* = .998), but with values significantly higher than the slices perfused only with Aβ_1–42_ (*p* = .008) that showed LTD instead of LTP (Figure 2a). However, the increase in GirK‐dependent signal with ML297 (10 μM) alone hindered LTP (*n* = 12 slices, Figure 2b,c, yellow diamonds and bar, respectively; 100.5 ± 1.8% of baseline*; p* = .059) probably because of a massive hyperpolarization that does not allow NMDA activation needed for LTP induction. Thus, the activation of GirK channels is able to protect the mechanisms needed for LTP induction from the depressive Aβ_1–42_ action in our in vitro amyloidosis model.

Given that Aβ has been shown to alter GirK channels at the molecular (May et al., 2017; Mayordomo‐Cava et al., 2015) and synaptic (Nava‐Mesa et al., 2013) levels, we asked whether blocking of GirK‐dependent signaling with TQ would interfere with synaptic plasticity depression induced by Aβ_1–42_. As lack of GirK channels has been shown to be deleterious for cognition (Slesinger et al., 2015) we first evaluated the effect of TQ (0.5 μM) on LTP induction. In Figure 2b it can be observed that when TQ was perfused HFS was able to generate a significant potentiation of the synaptic responses in comparison to Aβ_1–42_, although it was significantly lower than control's (*n* = 13 slices, Figure 2b,c; white triangles and bar; 112 ± 1.3% of baseline, *post hoc *vs. control, *p* < .042). However, pre‐application of TQ did not prevent the depressant effect of Aβ_1–42_ on synaptic plasticity and LTD was induced (*n* = 10 slices, Figure 2b,c; green circles and bar; 91.5 ± 2.9% of the baseline). This value was significantly different from control vehicle values (*p* = .005), and equivalent to the slices treated with Aβ_1–42_ (*p* = .817) indicating that the blockage of GirK channels by TQ did not directly interfere with the mechanism by which Aβ_1–42_ transforms HFS‐induced LTP into LTD in our in vitro model of early amyloidosis.

Finally, we asked whether GirK channels blocking with TQ would obstruct the protective effect of ML297 on LTP mechanisms disrupted by Aβ_1–42_. Before baseline recordings, slices were sequentially perfused by TQ (0.5 μM), to block GirK channels, and then Aβ_1–42_ (0.5 μM), and ML297 (10 μM) for 10 min each. As shown in Figure 2b,c, HFS also induced LTD (*i.e*., depressed LTP to 89.6 ± 3.6% of baseline; *n* = 6 slices, purple circles and bar), with similar values to Aβ_1–42_ or TQ + Aβ_1–42_ groups, but significantly different to control slices (*post hoc *vs. control vehicle *p* = .012). These results indicate that GirK channels blocking by TQ did not interfere with Aβ_1–42_ action but avoided the ML297 conservative effects on LTP mechanisms in our in vitro model of amyloidosis.

### 3.3 Hippocampal GirK protein pattern is not affected by Aβ_1–42_ in slice preparation

Protein expression of GIRK1 and GIRK2 was assessed in hippocampus slices treated with Aβ_1–42_ for incubations of 30 and 120 min (Figure 3). Using western blotting, we found the expected band of 60 KDa for GIRK1 (Figure 3a), as well as the 45 KDa band for GIRK2 (Figure 3b). Our results show that Aβ_1–42_ did not induce significant changes on the GirK subunits analyzed at the protein expression level in our in vitro model of amyloidosis.

### 3.4 Hippocampal GirK activation counteracts memory deficits induced by Aβ_1–42_

LTP is proposed to be the functional correlate that underlies learning and memory processes in the hippocampus (Bliss, Collingridge, & Morris, 2007). LTD has also been shown to have a relevant role in hippocampal‐dependent learning (Kemp & Manahan‐Vaughan, 2007). As we found in vitro that LTP deficits induced by Aβ_1–42_ consisted on the actual induction of LTD and were compensated by enhancing GirK‐dependent signaling (Figure 2b), we asked whether these results might be extrapolated to an in vivo system. For that purpose, we assessed hippocampal‐dependent memory with an habituation test as LTD has been mainly related to habituation forms of and to be mainly associated to habituation forms of memory (Collingridge, Peineau, Howland, & Wang, 2010) and extinction of previous memories (Bliss et al., 2007; Malleret et al., 2010). However, before habituation memory testing, we confirmed that spontaneous activity recorded from hippocampal CA1 area and motor coordination were not affected by drugs *i.c.v.*‐injected in the present work, as it had been previously reported that intraperitoneal injection of ML297 significantly decreased mice general motor activity (Kaufmann, Romaine, & Days, 2013) (See Supporting Information Figures S3 and S4). For such objective, electrophysiological recordings from CA1 neurons in slices and in freely moving mice were obtained (Figure S3), and a rotating rod test was performed after *i.c.v.* injections (Figure S4). Results confirmed that at drug concentrations used in our experiments, no significant alterations were observed neither on CA1 spontaneous activity (both in vitro and in vivo*;* Figure S3) nor on locomotion (Figure S4). We then evaluated habituation, an elementary form of non‐associative hippocampal‐dependent learning (Collingridge et al., 2010; Leussis & Bolivar, 2006) in our experimental groups. We challenged control (vehicle), Aβ_1–42_ and Aβ_1–42_ + ML297 *i.c.v.‐*treated groups on the Open Field Habituation Test performed in automatic *Laboratory Animal Behavior, Observation, Registration, and Analysis System* (LABORAS®). The task relies upon the tendency of rodents to decrease in exploratory behavior (exploratory habituation) in response to continued or repeated exposure to a novel environment as an open field. In the acquisition or training trial (Open field 1, OF1), there were no significant differences between traveled distances between mice assigned to each experimental group (Figure 4c,d; *n* = 31 animals; *F*_(2,28)_ = 2.51, *p* = .099). However, 24 hr later on the retention day (habituation session or OF2), exploratory movements were significantly reduced in all animals when compared with the training session (Figure 4c,d), indicating they all could recall the arena (vehicle: *t*(9) = −5.56, *p *= .0003; Aβ_1–42_: *t*(10) = −3.11; *p* = .011; Aβ_1–42_ *+ *ML297: *t*(9) = −4.63; *p* = .001). Moreover, the decrease in exploration was less noticeable for Aβ_1–42_ injected animals in comparison to controls (*post hoc* Aβ_1–42_ versus*.* vehicle: *p* = .037), showing that Aβ disrupted hippocampal‐dependent habituation in these animals (Figure 4c,d) and they could not remember the open field as well as controls. On the contrary, for Aβ_1–42_ + ML297‐treated mice levels of habituation (measured as exploration distance) were similar to controls (Figure 4c,d; *post hoc* Aβ_1–42_ + ML297 versus*.* vehicle: *p* = .929). Finally, in order to confirm our findings, and further discard any possible locomotion impairments induced by GirK modulation (Kaufmann et al., 2013), animals *i.c.v.‐*injected with ML297 or TQ alone together with the other 3 experimental groups (vehicle, Aβ_1–42_ and Aβ_1–42_ + ML297) were challenged in an additional open field habituation test (see Supporting Information, Figure S2). Results showed that, when GirK channels were specifically modulated by increasing (ML297) or decreasing (TQ) their activity, animals presented disrupted habituation memory when compared to controls (vehicle)—that is, showed higher levels of exploration than controls (Figure S2b,c) indicating an incomplete memory of the open field— in agreement with LTP impairments observed in vitro for both groups. However, in accordance with results described in Figure 2, Aβ_1–42_ + ML297 recovered control (vehicle) values of exploration levels previously increased by Aβ_1–42_ alone (see Figure S2b,c for details), confirming fully recall of the arena and therefore the interest of GirK channels modulation in amyloidosis models.

### 3.5 Hippocampal GirK protein expression is altered by Aβ_1–42_ in vivo

In order to assess whether GirK channels are targeted by Aβ_1–42_, we analyzed in our experimental groups the optical density of GIRK1 subunit immunostained sections through the *stratum lacunosum‐moleculare* of the dorsal hippocampus. GIRK 1 subunit analysis was chosen as ML297 and TQ modulate the activity of GIRK1‐containing channels (Wydeven et al., 2014; Yow et al., 2011). Our results show that *i.c.v.* injections of TQ produced a significant increase in GIRK1 staining (*n* = 16 slices, 112 ± 4.1% of control vehicle values; *post hoc *vs. control, *p *= .033). However, ML297 significantly decreased GIRK1 optical density (*n* = 15 slices, 78 ± 3.7% of control values, *post hoc *vs. control vehicle, *p* < .001), suggesting that both, pharmacological activation or blockage of GirK‐dependent signaling in the dorsal hippocampus have significant effect on channel protein expression. On the other hand, GIRK1 immunostaining was statistically lower in Aβ_1–42_‐injected animals in comparison with controls (*n* = 23 slices, 71 ± 3.4% of control values, *post hoc *vs. control, *p* < .001). Furthermore, ML297‐increased GirK channel activity was not able to prevent the down‐regulation induced by Aβ_1–42_ on GIRK1 subunit analyzed in Aβ_1–42_ + ML297‐treated mice (*n* = 25 slices, 70 ± 4.9% of control values, *post hoc *vs. control, *p* < .001) and showed similar optical density values than mice injected with Aβ_1–42_ alone (*post hoc *vs. Aβ_1–42_, *p *= .800). In summary, our data show that in vivo, GIRK1 subunit expression in the dorsal hippocampus is not only pharmacologically regulated by specific GirK drugs, but also down‐regulated by Aβ_1–42_ action.

## 4 DISCUSSION

Synaptic plasticity, the cellular substrate of learning in the hippocampus, has been shown to be altered in in vitro and in vivo models of AD (Styr & Slutsky, 2018; Tu, Okamoto, Lipton, & Xu, 2014) as the correct functionality of hippocampal neuronal circuits depends on a delicate balance between excitatory and inhibitory synaptic transmission, which is early affected by the action of Aβ (Busche & Konnerth, 2016; Pini et al., 2016; Villette & Dutar, 2017). The soluble forms of Aβ_1–42_ peptide are pathological species present in AD that are widely used to investigate their contribution to the pathogenesis and progression of the disease (Selkoe & Hardy, 2016). In vitro acute application of Aβ_1–42_ causes hippocampal hyperexcitability (Tamagnini et al., 2015; Varga et al., 2014) and deficits in LTP (Eslami et al., 2018; Kimura et al., 2012; Lei, Xu, & Li, 2016). Similarly, different in vivo experiments show that the administration of Aβ_1–42_ triggers behavioral deficits because of alterations in neural excitability and LTP process (Kalweit et al., 2015; Sanchez‐Rodriguez et al., 2019, 2017). In this regard, it is widely accepted that hyperexcitability induced by Aβ in the early stages of AD has a main role in plasticity alteration, although the mechanisms underlying such disruptions are not clear. In fact, controlling the excess of neuronal activity contributes to restore the excitation‐inhibition balance lost in AD brain (Huang & Mucke, 2012; Sanchez et al., 2012; Styr & Slutsky, 2018). Hence, GirK channels, as one of the main determinants for the resting membrane potential, might be able to compensate the hyperexcitability induced by Aβ in early stages of AD as already suggested (Nava‐Mesa et al., 2014). Our data show that Aβ switched hippocampal HFS‐induced LTP into LTD inducing memory deficits, effects that were avoided by GirK channels activation. These results suggest that LTP can be preserved from the deleterious action of Aβ by enhancing GirK activity and therefore support the interest of modulating GirK channels in pathologies where hyperexcitability is a hallmark.

### 4.1 Aβ_1–42_‐induced LTD by HFS protocol

One of the main results reported here is that, instead of LTP, Aβ_1–42_ induced LTD of the synaptic response recorded from CA1 region after HFS was applied at *Schaffer* collaterals. These results were previously observed in vivo, where LTP was abolished and showed a tendency to maintain depression of the synaptic response that lasted several days (Sanchez‐Rodriguez et al., 2017). Because of the role of GirK channels in maintaining a correct inhibitory tone in the hippocampus, it has been shown that their activity modulation modifies the threshold of stimulation necessary for NMDA receptors (NMDARs) activation and LTP induction (Malik & Johnston, 2017). It has been previously proposed that the molecular mechanism mediated by the activation of NMDARs may also produce an increase in the expression and activity of GirK channels (Chung, Ge, et al., 2009a; Chung, Qian, Ehlers, Jan, & Jan, 2009b). Thus, the HFS protocol applied in our experiments could temporarily modify the activity of these channels, as it has been already found within 15 min after NMDAR activation, with an increase in the surface density of GirK channels in soma, dendrites, and some spines of hippocampal neurons (Chung, Qian, et al., 2009b). Therefore, NMDAR‐induced GIRK surface expression might also modulate the threshold of stimulation necessary for NMDARs activation in LTP found in our in vitro experiments (Chung, Ge, et al., 2009a; Huang et al., 2005), as well as it has been recently shown in vivo (Sanchez‐Rodriguez et al., 2019). In fact, it has been reported that LTD may take place when threshold of LTP is not reached because stimulation fails to activate properly the post‐synaptic neurons. This situation can be explained by the Bienenstock, Cooper and Munro (BCM) theory of synaptic plasticity that proposes that a certain threshold is needed for LTP induction (Cooper & Bear, 2012). Therefore, Aβ_1–42_ might decrease the levels of response to HFS (*i.e*., below the threshold) inducing LTD instead of LTP. This interesting mechanism has also been reported in basolateral amygdaloid nucleus‐insular cortex projection where, as in our experiments, Aβ_1–42_ has shown to shift HFS‐induced LTP to LTD (Moreno‐Castilla et al., 2016). These authors found Aβ_1–42_ to modify the threshold for the induction of cortical LTP and/or LTD through dopaminergic neurotransmission, in agreement with BCM theory. These findings suggest the importance of maintaining the correct neural excitability levels to preserve physiological synaptic plasticity processes in amyloidosis models.

In the hippocampus LTD is normally induced by NMDARs stimulation in response to low‐frequency stimulation protocols (Bliss et al., 2007). LTP and LTD share the same activation pathways through NMDARs, and the induction of one or the other depends on subtle changes in the Ca^2+^ concentration (Lisman, 1989) that, according to the BCM theory, could govern the LTP induction threshold (Cooper & Bear, 2012). Higher increases produce LTP, whereas discrete ones facilitate LTD. HFS in normal conditions activates NMDARs and increases Ca^2+^ influx, inducing LTP. Therefore, the induction of LTD might be because of a decrease in Ca^2+^ influx through NMDARs. Indeed, it has been reported that Aβ_1–42_ alters NMDAR function by decreasing Ca^2+^ influx and facilitates LTD (Li et al., 2009, 2011). Hence, NMDARs emerge as a pivotal key in the threshold for the determination of the synaptic plasticity direction (Bliss et al., 2007; Cooper & Bear, 2012; Lisman, 1989). Indeed, to explain the increased neural hyperexcitability in acute models of AD, Aβ has also been proposed to act through NMDA receptor (NMDAR) (Hsieh et al., 2006; Rammes et al., 2018). Since glutamate has a double role as excitatory neurotransmitter and as precursor for GABA, it would be responsible for altering the neurotransmission balance that contributes to plasticity impairments in AD (Palop & Mucke, 2010).

### 4.2 LTP and LTD in learning and memory

In addition to LTP (Bliss et al., 2007), LTD has also been proposed to play an important role in hippocampal‐dependent learning (Kemp & Manahan‐Vaughan, 2007) and to be mainly associated to habituation forms of memory (Collingridge et al., 2010) as well as clearing of old memory traces (Bliss et al., 2007; Malleret et al., 2010). In agreement with our data, Aβ_1–42_ has been previously shown to facilitate LTD (Li et al., 2009; Renner et al., 2010) through glutamatergic neurotransmission. Such augmented LTD at Schaffer collateral–CA1 synapses, as HFS‐induced LTD produced by Aβ_1–42_ reported here, has been proposed as an underlying mechanism for deficits in habituation to a novel environment and acquisition of hippocampus‐dependent memory tasks (Zeng et al., 2001). Thus, Aβ_1–42_ might be inducing a significant shift in the LTP/LTD threshold that would explain our results in the habituation test, where animals *i*.*c*.*v*.‐injected with Aβ_1–42_ were not able to fully recall the open field on the retention day. Similar results have been reported for novel object recognition test (Sanchez‐Rodriguez et al., 2017), a memory task that also depends on hippocampal LTP (Clarke, Cammarota, Gruart, Izquierdo, & Delgado‐Garcia, 2010). In contrast, GirK activation opposed to the effect of Aβ_1–42_, enabling LTP induction and subsequent recovery of hippocampal‐dependent habituation memory in the open field habituation test. The results are in agreement with the contention that controlling neuronal hyperactivity reestablishes excitation‐inhibition levels and upstreaming functions lost in AD brain (Busche & Konnerth, 2015; Zott et al., 2018).

### 4.3 GirK modulation and hippocampal CA3‐CA1 LTP restoration

We also asked for the mechanism by which an increase in GirK conductance could compensate the excess of neuronal excitability caused by Aβ (Nava‐Mesa et al., 2013; Tamagnini et al., 2015; Varga et al., 2014) and therefore, restore hippocampal synaptic plasticity mechanisms. Present data showed that ML297 was also able to preserve mechanisms needed for LTP induction from the deleterious effects of Aβ_1–42_, most likely by recovering the balance between excitatory and inhibitory neurotransmission in CA3—CA1 synapse acting mainly at post‐synaptic level on pyramidal cells, as we also reported in vivo (Sanchez‐Rodriguez et al., 2017). But Aβ_1–42_ transformed HFS‐induced LTP into LTD even when GirK channels were previously blocked by the specific blocker TQ. Moreover, if TQ was present, activation with ML297 failed to restore the LTP switched into LTD by Aβ_1–42_ at the CA3—CA1 hippocampal synapse. One possible explanation for these results might be that ML297 produces GirK activation through direct binding to the amino acid F137 of the GIRK1‐subunit (Wydeven et al., 2014). However, TQ action has been associated to the interaction with GIRK4 subunit, more than GIRK1 (Yow et al., 2011), although these experiments were carried out using GIRK1 mutant homotetramers (KIR3.1^F137S^). As TQ has been reported to block GIRK1/2 channels (Kanjhan, Coulson, Adams, & Bellingham, 2005), F137 is likely essential as binding site of GirK channels also for TQ. This situation would establish a competitive binding in which ML297 would not be able to increase GirK channel conductance in the presence of TQ, explaining why TQ prevented the restoration of LTP by ML297 in our in vitro model of AD. Our results indicate that regardless the mechanism by which Aβ_1–42_ induces hyperexcitability, increasing GirK‐dependent signaling is able to compensate alterations caused by Aβ_1–42_ on LTP process.

### 4.4 GirK channels and Aβ

Despite the apparent causal relationship of Aβ in the pathogenesis of AD, its mechanisms of action or main targets have not yet been established (Selkoe & Hardy, 2016). Previous experiments have showed that incubation of rat hippocampal slices with Aβ_25–35_ produces a facilitation in LFS‐induced LTD (Cheng, Yin, Zhang, & Qi, 2009), causes a decrease in the expression of the Girk1‐4 subunits at the mRNA level (Mayordomo‐Cava et al., 2015) and, as TQ, a membrane depolarization and synaptic neurotransmission impairment by a reduction in GirK channels conductance (Nava‐Mesa et al., 2013). Aβ_25–35_, the active fragment of Aβ peptide, and Aβ_1–42_, a more relevant specie in AD patients, seem to share neurophysiopathological mechanisms as both induce an imbalance in the excitatory/inhibitory neurotransmission in the hippocampus that causes aberrant activity (Gutierrez‐Lerma, Ordaz, & Pena‐Ortega, 2013). In addition, pathological GirK channel activity induced by Aβ_1–42_ has been related to neuronal degeneration and apoptosis mechanisms (May et al., 2017). These data suggest GirK as one of the putative targets on which Aβ could be acting on. However, in this study, we found that in hippocampal slices incubated with Aβ_1–42_, protein expression pattern for GIRK1 and 2 subunits did not change. It has been reported that excitotoxic oligomeric Aβ_1–42_ may cause a rapid redistribution of existing GirK subunits to the membrane surface (May et al., 2017), which could explain that protein expression did not seem to change. On the other hand, the immunohistochemical analysis of GIRK1 subunit in the dorsal hippocampus of our different experimental groups showed a significant modulation of GIRK1 protein expression levels, increasing when channel is blocked by TQ, whereas decreasing when opened by ML297, as expected for neuronal drug sensitization and desensitization processes, respectively (Golan, Armstrong, & A.W., A., 2016). We also found a significant decrease in GIRK1 subunit induced by Aβ_1–42_ injection that was not reversed by the addition of ML297. This result strongly suggests a link between Aβ_1–42_ and GirK channels at least in the dorsal hippocampus, which could very likely be masked in western blot analysis by all other regions included in the hippocampal slice but, on the contrary, be detected at the gen level by a higher sensitive method such as RT‐qPCR (Mayordomo‐Cava et al., 2015). The decrease in GirK expression found in the present work would be in agreement with the increase in hyperexcitability observed in vivo in the dorsal hippocampus (Sanchez‐Rodriguez et al., 2017), as well as with the hippocampal‐dependent memory recovery produced by GirK activation with ML297 when combined with Aβ_1–42_ in the present work. Additionally, it is also important to note the temporal course of the experiments. Our data indicate that Aβ_1–42_ might induce GirK channels to decrease in the long‐term, a mechanism similar to sensitization process. Then, Aβ_1–42_ modulation of GirK channel might be found analyzing specific regions of the hippocampus (immunohistochemical approach), but it could not be observed in the hippocampus as a whole (western blot analysis). Nevertheless, present data suggest that GirK channels can be taken into account to explain some of the effects of Aβ_1–42_ (May et al., 2017; Nava‐Mesa et al., 2013; Sanchez‐Rodriguez et al., 2019, 2017).

## 5 CONCLUSIONS

The finding that hyperexcitability could be an early neuronal dysfunction in AD (Busche & Konnerth, 2015; Palop & Mucke, 2009) has had important consequences for AD research field in the last decade. Consistent with this concept, antiepileptic drugs mainly targeted on the glutamatergic system, such as levetiracetam (Sanchez et al., 2012), have been used to compensate the excess of excitation, although with limited success (Vossel et al., 2017). Consequently, inhibitory neurotransmission systems have gained great interest in recent years, not only as potential pharmacological targets, but also as part of combinational therapies that might synergistically protect neuronal degradation in AD (Calvo‐Flores Guzman et al., 2018; Nava‐Mesa et al., 2014). One interesting example is the combined drug therapy using the NMDA‐receptor antagonist, acamprosate and the GABA_B_ agonist, baclofen (Hill & Bowery, 1981) whose main effector is the GirK channel, with significant benefits over monotherapeutics (Chumakov et al., 2015). With this scenario, our current data highlight the importance of understanding how modulation of GirK channels, as the main effectors of many inhibitory receptors, contribute to preserve synaptic plasticity mechanisms from the deleterious effects of Aβ.

## CONFLICT OF INTEREST

None of the authors has a conflict of interest.

### Open Science Badges

This article has received a badge for *Open Materials* because it provided all relevant information to reproduce the study in the manuscript. More information about the Open Science badges can be found at https://cos.io/our-services/open-science-badges/

## References

[jnc14946-bib-0001] Bakker, A. , Krauss, G. L. , Albert, M. S. , Speck, C. L. , Jones, L. R. , Stark, C. E. , … Gallagher, M. (2012). Reduction of hippocampal hyperactivity improves cognition in amnestic mild cognitive impairment. Neuron, 74, 467–474. 10.1016/j.neuron.2012.03.023 22578498 PMC3351697

[jnc14946-bib-0002] Bliss, T. V. , Collingridge, G. L. , & Morris, R. (2007). Synaptic Plasticity in the Hippocampus In Andersen P., Morris R., Amaral D. G., Bliss T., & J. O´Keefe, (Eds.), The Hippocampus Book (pp. 343–474). New York: Oxford University Press.

[jnc14946-bib-0003] Bliss, T. , Collingridge, G. L. , Morris, R. , & Reymann, K. G. (2018). Long‐term potentiation in the hippocampus: Discovery, mechanisms and function. Neuroforum, 24, A103–A120. 10.1515/nf-2017-A059

[jnc14946-bib-0004] Busche, M. A. , & Konnerth, A. (2015). Neuronal hyperactivity–A key defect in Alzheimer's disease? BioEssays, 37, 624–632. 10.1002/bies.201500004 25773221

[jnc14946-bib-0005] Busche, M. A. , & Konnerth, A. (2016). Impairments of neural circuit function in Alzheimer's disease (p. 371). Sci: Philos. Trans. R. Soc. Lond B Biol. 10.1098/rstb.2015.0429 PMC4938029 27377723

[jnc14946-bib-0006] Calvo‐Flores Guzman, B. , Vinnakota, C. , Govindpani, K. , Waldvogel, H. J. , Faull, R. L. M. , & Kwakowsky, A. (2018). The GABAergic system as a therapeutic target for Alzheimer's disease. Journal of Neurochemistry, 146, 649–669. 10.1111/jnc.14345 29645219

[jnc14946-bib-0007] Cheng, L. , Yin, W. J. , Zhang, J. F. , & Qi, J. S. (2009). Amyloid beta‐protein fragments 25–35 and 31–35 potentiate long‐term depression in hippocampal CA1 region of rats in vivo. Synapse (New York, N. Y.), 63, 206–214. 10.1002/syn.20599 19072840

[jnc14946-bib-0008] Chumakov, I. , Nabirotchkin, S. , Cholet, N. , Milet, A. , Boucard, A. , Toulorge, D. , … Cohen, D. (2015). Combining two repurposed drugs as a promising approach for Alzheimer's disease therapy. Scientific Reports, 5, 7608 10.1038/srep07608 25566747 PMC5378993

[jnc14946-bib-0009] Chung, H. J. , Ge, W. P. , Qian, X. , Wiser, O. , Jan, Y. N. , & Jan, L. Y. (2009a). G protein‐activated inwardly rectifying potassium channels mediate depotentiation of long‐term potentiation. Proc Natl Acad Sci U S A, 106, 635–640. 10.1073/pnas.0811685106 19118199 PMC2613041

[jnc14946-bib-0010] Chung, H. J. , Qian, X. , Ehlers, M. , Jan, Y. N. , & Jan, L. Y. (2009b). Neuronal activity regulates phosphorylation‐dependent surface delivery of G protein‐activated inwardly rectifying potassium channels. Proc Natl Acad Sci U S A, 106, 629–634. 10.1073/pnas.0811615106 19118198 PMC2613039

[jnc14946-bib-0011] Clarke, J. R. , Cammarota, M. , Gruart, A. , Izquierdo, I. , & Delgado‐Garcia, J. M. (2010). Plastic modifications induced by object recognition memory processing. Proceedings of the National Academy of Sciences of the United States of America, 107, 2652–2657. 10.1073/pnas.0915059107 20133798 PMC2823877

[jnc14946-bib-0012] Collingridge, G. L. , Peineau, S. , Howland, J. G. , & Wang, Y. T. (2010). Long‐term depression in the CNS. Nature Reviews Neuroscience, 11, 459–473. 10.1038/nrn2867 20559335

[jnc14946-bib-0013] Cooper, L. N. , & Bear, M. F. (2012). The BCM theory of synapse modification at 30: Interaction of theory with experiment. Nature Reviews Neuroscience, 13, 798–810. 10.1038/nrn3353 23080416

[jnc14946-bib-0014] Drake, C. T. , Bausch, S. B. , Milner, T. A. , & Chavkin, C. (1997). GIRK1 immunoreactivity is present predominantly in dendrites, dendritic spines, and somata in the CA1 region of the hippocampus. Proceedings of the National Academy of Sciences of the United States of America, 94, 1007–1012. 10.1073/pnas.94.3.1007 9023373 PMC19630

[jnc14946-bib-0015] Eslami, M. , Sadeghi, B. , & Goshadrou, F. (2018). Chronic ghrelin administration restores hippocampal long‐term potentiation and ameliorates memory impairment in rat model of Alzheimer's disease. Hippocampus. 10.1002/hipo.23002 30009391

[jnc14946-bib-0016] Fernandez‐Alacid, L. , Watanabe, M. , Molnar, E. , Wickman, K. , & Lujan, R. (2011). Developmental regulation of G protein‐gated inwardly‐rectifying K+ (GIRK/Kir3) channel subunits in the brain. European Journal of Neuroscience, 34, 1724–1736. 22098295 10.1111/j.1460-9568.2011.07886.x PMC3936682

[jnc14946-bib-0017] Glaaser, I. W. , & Slesinger, P. A. (2015). Structural Insights into GIRK Channel Function. International Review of Neurobiology, 123, 117–160. 26422984 10.1016/bs.irn.2015.05.014

[jnc14946-bib-0018] Golan, D. A. , Armstrong, E. J. , & A.W., A., (2016). Principles of Pharmacology : The Pathophysiologic Basis of Drug Therapy. Philadelphia: Lippincott Williams and Wilkins.

[jnc14946-bib-0019] Goutagny, R. , & Krantic, S. (2013). Hippocampal oscillatory activity in Alzheimer's disease: Toward the identification of early biomarkers? Aging Dis., 4, 134–140. 23730529 PMC3660123

[jnc14946-bib-0020] Gutierrez‐Lerma, A. I. , Ordaz, B. , & Pena‐Ortega, F. (2013). Amyloid Beta peptides differentially affect hippocampal theta rhythms in vitro. Int. J. Pept., 2013, 328140. 23878547 10.1155/2013/328140 PMC3708430

[jnc14946-bib-0021] Hill, D. R. , & Bowery, N. G. (1981). 3H‐baclofen and 3H‐GABA bind to bicuculline‐insensitive GABA B sites in rat brain. Nature, 290, 149–152. 10.1038/290149a0 6259535

[jnc14946-bib-0022] Hsieh, H. , Boehm, J. , Sato, C. , Iwatsubo, T. , Tomita, T. , Sisodia, S. , & Malinow, R. (2006). AMPAR removal underlies Abeta‐induced synaptic depression and dendritic spine loss. Neuron, 52, 831–843. 17145504 10.1016/j.neuron.2006.10.035 PMC1850952

[jnc14946-bib-0023] Huang, C. S. , Shi, S. H. , Ule, J. , Ruggiu, M. , Barker, L. A. , Darnell, R. B. , … Jan, L. Y. (2005). Common molecular pathways mediate long‐term potentiation of synaptic excitation and slow synaptic inhibition. Cell, 123, 105–118. 10.1016/j.cell.2005.07.033 16213216

[jnc14946-bib-0024] Huang, Y. , & Mucke, L. (2012). Alzheimer mechanisms and therapeutic strategies. Cell, 148, 1204–1222. 10.1016/j.cell.2012.02.040 22424230 PMC3319071

[jnc14946-bib-0025] Jan, A. , Hartley, D. M. , & Lashuel, H. A. (2010). Preparation and characterization of toxic Abeta aggregates for structural and functional studies in Alzheimer's disease research. Nature Protocols, 5, 1186–1209. 20539293 10.1038/nprot.2010.72

[jnc14946-bib-0026] Kalweit, A. N. , Yang, H. , Colitti‐Klausnitzer, J. , Fulop, L. , Bozso, Z. , Penke, B. , & Manahan‐Vaughan, D. (2015). Acute intracerebral treatment with amyloid‐beta (1–42) alters the profile of neuronal oscillations that accompany LTP induction and results in impaired LTP in freely behaving rats. Frontiers in Behavioural Neurosciences, 9, 103. 10.3389/fnbeh.2015.00103 PMC4422036 25999827

[jnc14946-bib-0027] Kanjhan, R. , Coulson, E. J. , Adams, D. J. , & Bellingham, M. C. (2005). Tertiapin‐Q blocks recombinant and native large conductance K+ channels in a use‐dependent manner. Journal of Pharmacology and Experimental Therapeutics, 314, 1353–1361. 15947038 10.1124/jpet.105.085928

[jnc14946-bib-0028] Kaufmann, K. , Romaine, I. , Days, E. *et al* (2013). ML297 (VU0456810), the first potent and selective activator of the GIRK potassium channel, displays antiepileptic properties in mice. ACS Chem. Neurosci., 4, 1278–1286. 23730969 10.1021/cn400062a PMC3778424

[jnc14946-bib-0029] Kemp, A. , & Manahan‐Vaughan, D. (2007). Hippocampal long‐term depression: Master or minion in declarative memory processes? Trends in Neurosciences, 30, 111–118. 10.1016/j.tins.2007.01.002 17234277

[jnc14946-bib-0030] Kim, C. S. , & Johnston, D. (2015). A1 adenosine receptor‐mediated GIRK channels contribute to the resting conductance of CA1 neurons in the dorsal hippocampus. Journal of Neurophysiology, 113, 2511–2523. 10.1152/jn.00951.2014 25652929 PMC4416607

[jnc14946-bib-0031] Kimura, R. , MacTavish, D. , Yang, J. , Westaway, D. , & Jhamandas, J. H. (2012). Beta amyloid‐induced depression of hippocampal long‐term potentiation is mediated through the amylin receptor. Journal of Neuroscience, 32, 17401–17406. 10.1523/JNEUROSCI.3028-12.2012 23197731 PMC6621862

[jnc14946-bib-0032] Kotecki, L. , Hearing, M. , McCall, N. M. , Marron Fernandez de Velasco, E. , Pravetoni, M. , Arora, D. , … Wickman, K. (2015). GIRK Channels Modulate Opioid‐Induced Motor Activity in a Cell Type‐ and Subunit‐Dependent Manner. Journal of Neuroscience, 35, 7131–7142. 10.1523/JNEUROSCI.5051-14.2015 25948263 PMC4420781

[jnc14946-bib-0033] Kumar, A. (2011). Long‐Term Potentiation at CA3‐CA1 Hippocampal Synapses with Special Emphasis on Aging, Disease, and Stress. Frontiers in Aging Neuroscience, 3, 7 10.3389/fnagi.2011.00007 21647396 PMC3102214

[jnc14946-bib-0034] Kurudenkandy, F. R. , Zilberter, M. , Biverstal, H. , Presto, J. , Honcharenko, D. , Stromberg, R. , … Fisahn, A. (2014). Amyloid‐beta‐induced action potential desynchronization and degradation of hippocampal gamma oscillations is prevented by interference with peptide conformation change and aggregation. Journal of Neuroscience, 34, 11416–11425. 25143621 10.1523/JNEUROSCI.1195-14.2014 PMC6615507

[jnc14946-bib-0035] Lei, M. , Xu, H. , Li, Z. *et al* (2016). Soluble Abeta oligomers impair hippocampal LTP by disrupting glutamatergic/GABAergic balance. Neurobiology of Diseases, 85, 111–121. 10.1016/j.nbd.2015.10.019 PMC4778388 26525100

[jnc14946-bib-0036] Leussis, M. P. , & Bolivar, V. J. (2006). Habituation in rodents: A review of behavior, neurobiology, and genetics. Neuroscience and Biobehavioral Reviews, 30, 1045–1064. 10.1016/j.neubiorev.2006.03.006 16774787

[jnc14946-bib-0037] Li, S. , Hong, S. , Shepardson, N. E. , Walsh, D. M. , Shankar, G. M. , & Selkoe, D. (2009). Soluble oligomers of amyloid Beta protein facilitate hippocampal long‐term depression by disrupting neuronal glutamate uptake. Neuron, 62, 788–801. 19555648 10.1016/j.neuron.2009.05.012 PMC2702854

[jnc14946-bib-0038] Li, S. , Jin, M. , Koeglsperger, T. , Shepardson, N. E. , Shankar, G. M. , & Selkoe, D. J. (2011). Soluble Abeta oligomers inhibit long‐term potentiation through a mechanism involving excessive activation of extrasynaptic NR2B‐containing NMDA receptors. Journal of Neuroscience, 31, 6627–6638. 21543591 10.1523/JNEUROSCI.0203-11.2011 PMC3100898

[jnc14946-bib-0039] Lisman, J. (1989). A mechanism for the Hebb and the anti‐Hebb processes underlying learning and memory. Proc Natl Acad Sci U S A, 86, 9574–9578. 10.1073/pnas.86.23.9574 2556718 PMC298540

[jnc14946-bib-0040] Lomo, T. (2018). Discovering long‐term potentiation (LTP) ‐ recollections and reflections on what came after. Acta Physiologica, Oxf, 222 10.1111/apha.12921 28719040

[jnc14946-bib-0041] Lujan, R. , & Aguado, C. (2015). Localization and Targeting of GIRK Channels in Mammalian Central Neurons. International Review of Neurobiology, 123, 161–200. 26422985 10.1016/bs.irn.2015.05.009

[jnc14946-bib-0042] Lujan, R. , Fernandez, M. , de Velasco, E. , Aguado, C. , & Wickman, K. (2014). New insights into the therapeutic potential of Girk channels. Trends in Neurosciences, 37, 20–29. 10.1016/j.tins.2013.10.006 24268819 PMC3880623

[jnc14946-bib-0043] Luscher, C. , & Malenka, R. C. (2012). NMDA receptor‐dependent long‐term potentiation and long‐term depression (LTP/LTD). Cold Spring Harb Perspect Biol, 4 10.1101/cshperspect.a005710 PMC3367554 22510460

[jnc14946-bib-0044] Luscher, C. , & Slesinger, P. A. (2010). Emerging roles for G protein‐gated inwardly rectifying potassium (GIRK) channels in health and disease. Nature Reviews Neuroscience, 11, 301–315. 20389305 10.1038/nrn2834 PMC3052907

[jnc14946-bib-0045] Malik, R. , & Johnston, D. (2017). Dendritic GIRK Channels Gate the Integration Window, Plateau Potentials, and Induction of Synaptic Plasticity in Dorsal But Not Ventral CA1 Neurons. Journal of Neuroscience, 37, 3940–3955. 10.1523/JNEUROSCI.2784-16.2017 28280255 PMC5394901

[jnc14946-bib-0046] Malleret, G. , Alarcon, J. M. , Martel, G. , Takizawa, S. , Vronskaya, S. , Yin, D. , … Shumyatsky, G. P. (2010). Bidirectional regulation of hippocampal long‐term synaptic plasticity and its influence on opposing forms of memory. Journal of Neuroscience, 30, 3813–3825. 10.1523/JNEUROSCI.1330-09.2010 20220016 PMC6632240

[jnc14946-bib-0047] May, L. M. , Anggono, V. , Gooch, H. M. , Jang, S. E. , Matusica, D. , Kerbler, G. M. , … Coulson, E. J. (2017). G‐Protein‐Coupled Inwardly Rectifying Potassium (GIRK) Channel Activation by the p75 Neurotrophin Receptor Is Required for Amyloid beta Toxicity. Front Neurosci, 11, 455. 28848381 10.3389/fnins.2017.00455 PMC5550722

[jnc14946-bib-0048] Mayordomo‐Cava, J. , Yajeya, J. , Navarro‐Lopez, J. D. , & Jimenez‐Diaz, L. (2015). Amyloid‐beta(25–35) Modulates the Expression of GirK and KCNQ Channel Genes in the Hippocampus. PLoS ONE, 10, e0134385. 26218288 10.1371/journal.pone.0134385 PMC4517786

[jnc14946-bib-0049] Moreno‐Castilla, P. , Rodriguez‐Duran, L. F. , Guzman‐Ramos, K. , Barcenas‐Femat, A. , Escobar, M. L. , & Bermudez‐Rattoni, F. (2016). Dopaminergic neurotransmission dysfunction induced by amyloid‐beta transforms cortical long‐term potentiation into long‐term depression and produces memory impairment. Neurobiology of Aging, 41, 187–199. 27103531 10.1016/j.neurobiolaging.2016.02.021

[jnc14946-bib-0050] Nava‐Mesa, M. O. , Jimenez‐Diaz, L. , Yajeya, J. , & Navarro‐Lopez, J. D. (2013). Amyloid‐beta induces synaptic dysfunction through G protein‐gated inwardly rectifying potassium channels in the fimbria‐CA3 hippocampal synapse. Front Cell Neurosci, 7, 117. 23898239 10.3389/fncel.2013.00117 PMC3722514

[jnc14946-bib-0051] Nava‐Mesa, M. O. , Jimenez‐Diaz, L. , Yajeya, J. , & Navarro‐Lopez, J. D. (2014). GABAergic neurotransmission and new strategies of neuromodulation to compensate synaptic dysfunction in early stages of Alzheimer's disease. Front Cell Neurosci, 8, 167. 24987334 10.3389/fncel.2014.00167 PMC4070063

[jnc14946-bib-0052] Palop, J. J. , & Mucke, L. (2009). Epilepsy and cognitive impairments in Alzheimer disease. Archives of Neurology, 66, 435–440. 19204149 10.1001/archneurol.2009.15 PMC2812914

[jnc14946-bib-0053] Palop, J. J. , & Mucke, L. (2010). Amyloid‐beta‐induced neuronal dysfunction in Alzheimer's disease: From synapses toward neural networks. Nature Neuroscience, 13, 812–818. 20581818 10.1038/nn.2583 PMC3072750

[jnc14946-bib-0054] Palop, J. J. , & Mucke, L. (2016). Network abnormalities and interneuron dysfunction in Alzheimer disease. Nature Reviews Neuroscience, 17, 777–792. 27829687 10.1038/nrn.2016.141 PMC8162106

[jnc14946-bib-0055] Paxinos, G. , & Franklin, K. B. (2001). The Mouse Brain in Stereotaxic Coordinates. London: Academic Press.

[jnc14946-bib-0056] Pini, L. , Pievani, M. , Bocchetta, M. , Altomare, D. , Bosco, P. , Cavedo, E. , … Frisoni, G. B. (2016). Brain atrophy in Alzheimer's Disease and aging. Ageing Research Reviews, 30, 25–48. 10.1016/j.arr.2016.01.002 26827786

[jnc14946-bib-0057] Rammes, G. , Seeser, F. , Mattusch, K. , Zhu, K. , Haas, L. , Kummer, M. , … Parsons, C. G. (2018). The NMDA receptor antagonist Radiprodil reverses the synaptotoxic effects of different amyloid‐beta (Abeta) species on long‐term potentiation (LTP). Neuropharmacology, 140, 184–192. 30016667 10.1016/j.neuropharm.2018.07.021

[jnc14946-bib-0058] Renner, M. , Lacor, P. N. , Velasco, P. T. , Xu, J. , Contractor, A. , Klein, W. L. , & Triller, A. (2010). Deleterious effects of amyloid beta oligomers acting as an extracellular scaffold for mGluR5. Neuron, 66, 739–754. 20547131 10.1016/j.neuron.2010.04.029 PMC3111138

[jnc14946-bib-0059] Sanchez, P. E. , Zhu, L. , Verret, L. , Vossel, K. A. , Orr, A. G. , Cirrito, J. R. , … Mucke, L. (2012). Levetiracetam suppresses neuronal network dysfunction and reverses synaptic and cognitive deficits in an Alzheimer's disease model. Proceedings of the National Academy of Sciences of the United States of America, 109, E2895–E2903. 10.1073/pnas.1121081109 22869752 PMC3479491

[jnc14946-bib-0060] Sanchez‐Rodriguez, I. , Gruart, A. , Delgado‐Garcia, J. M. , Jimenez‐Diaz, L. , & Navarro‐Lopez, J. D. (2019) Role of GirK channels in long‐term potentiation of synaptic inhibition in an in vivo mouse model of early amyloid‐beta pathology. International Journal of Molecular Sciences, 20, 1168. 10.3390/ijms20051168 PMC6429279 30866445

[jnc14946-bib-0061] Sanchez‐Rodriguez, I. , Temprano‐Carazo, S. , Najera, A. , Djebari, S. , Yajeya, J. , Gruart, A. , … Navarro‐Lopez, J. D. (2017). Activation of G‐protein‐gated inwardly rectifying potassium (Kir3/GirK) channels rescues hippocampal functions in a mouse model of early amyloid‐beta pathology. Scientific Reports, 7, 14658. 29116174 10.1038/s41598-017-15306-8 PMC5676742

[jnc14946-bib-0062] Schuette, S. R. , Fernandez‐Fernandez, D. , Lamla, T. , Rosenbrock, H. , & Hobson, S. (2016). Overexpression of protein kinase mzeta in the hippocampus enhances long‐term potentiation and long‐term contextual but not cued fear memory in rats. Journal of Neuroscience, 36, 4313–4324. 27076427 10.1523/JNEUROSCI.3600-15.2016 PMC4829652

[jnc14946-bib-0063] Selkoe, D. J. , & Hardy, J. (2016). The amyloid hypothesis of Alzheimer's disease at 25 years. EMBO Molecular Medicine, 8, 595–608. 27025652 10.15252/emmm.201606210 PMC4888851

[jnc14946-bib-0064] Slesinger, P. A. (2015). Wickman and K. Structure to Function of G protein‐gated inwardly rectifying (GIRK) channels. International Review of Neurobiology. Elsevier Inc.

[jnc14946-bib-0065] Styr, B. , & Slutsky, I. (2018). Imbalance between firing homeostasis and synaptic plasticity drives early‐phase Alzheimer's disease. Nature Neuroscience, 21, 463–473. 10.1038/s41593-018-0080-x 29403035 PMC6533171

[jnc14946-bib-0066] Tamagnini, F. , Scullion, S. , Brown, J. T. , & Randall, A. D. (2015). Intrinsic excitability changes induced by acute treatment of hippocampal CA1 pyramidal neurons with exogenous amyloid beta peptide. Hippocampus, 25, 786–797. 25515596 10.1002/hipo.22403 PMC4791149

[jnc14946-bib-0067] Teplow, D. B. (2006). Preparation of amyloid beta‐protein for structural and functional studies. Methods in Enzymology, 413, 20–33. 17046389 10.1016/S0076-6879(06)13002-5

[jnc14946-bib-0068] Tu, S. , Okamoto, S. , Lipton, S. A. , & Xu, H. (2014). Oligomeric Abeta‐induced synaptic dysfunction in Alzheimer's disease. Molecular Neurodegeneration, 9, 48. 25394486 10.1186/1750-1326-9-48 PMC4237769

[jnc14946-bib-0069] Ulrich, D. (2015). Amyloid‐beta impairs synaptic inhibition via GABA(A) receptor endocytosis. Journal of Neuroscience, 35, 9205–9210. 26085642 10.1523/JNEUROSCI.0950-15.2015 PMC6605158

[jnc14946-bib-0070] Varga, E. , Juhasz, G. , Bozso, Z. , Penke, B. , Fulop, L. , & Szegedi, V. (2014). Abeta(1–42) enhances neuronal excitability in the CA1 via NR2B subunit‐containing NMDA receptors. Neural Plasticity, 2014, 584314 10.1155/2014/584314 25276438 PMC4168240

[jnc14946-bib-0071] Varga, E. , Juhasz, G. , Bozso, Z. , Penke, B. , Fulop, L. , & Szegedi, V. (2015). Amyloid‐beta1‐42 disrupts synaptic plasticity by altering glutamate recycling at the synapse. Journal of Alzheimer's Disease, 45, 449–456. 10.3233/JAD-142367 25547631

[jnc14946-bib-0072] Vega‐Avelaira, D. , Moss, A. , & Fitzgerald, M. (2007). Age‐related changes in the spinal cord microglial and astrocytic response profile to nerve injury. Brain, Behavior, and Immunity, 21, 617–623. 10.1016/j.bbi.2006.10.007 17158026

[jnc14946-bib-0073] Villette, V. , & Dutar, P. (2017). GABAergic microcircuits in Alzheimer's disease models. Current Alzheimer Research, 14, 30–39. 10.2174/1567205013666160819125757 27539596

[jnc14946-bib-0074] Vossel, K. A. , Tartaglia, M. C. , Nygaard, H. B. , Zeman, A. Z. , & Miller, B. L. (2017). Epileptic activity in Alzheimer's disease: Causes and clinical relevance. The Lancet Neurology, 16, 311–322. 10.1016/S1474-4422(17)30044-3 28327340 PMC5973551

[jnc14946-bib-0075] Wydeven, N. , Fernandez, M. , de Velasco, E. , Du, Y. , Benneyworth, M. A. , Hearing, M. C. , … Wickman, K. (2014). Mechanisms underlying the activation of G‐protein‐gated inwardly rectifying K^+^ (GIRK) channels by the novel anxiolytic drug, ML297. Proceedings of the National Academy of Sciences of the United States of America, 111, 10755–10760. 10.1073/pnas.1405190111 25002517 PMC4115558

[jnc14946-bib-0076] Yow, T. T. , Pera, E. , Absalom, N. , Heblinski, M. , Johnston, G. A. , Hanrahan, J. R. , & Chebib, M. (2011). Naringin directly activates inwardly rectifying potassium channels at an overlapping binding site to tertiapin‐Q. British Journal of Pharmacology, 163, 1017–1033. 21391982 10.1111/j.1476-5381.2011.01315.x PMC3130948

[jnc14946-bib-0077] Zeng, H. , Chattarji, S. , Barbarosie, M. , Rondi‐Reig, L. , Philpot, B. D. , Miyakawa, T. , … Tonegawa, S. (2001). Forebrain‐specific calcineurin knockout selectively impairs bidirectional synaptic plasticity and working/episodic‐like memory. Cell, 107, 617–629. 10.1016/S0092-8674(01)00585-2 11733061

[jnc14946-bib-0078] Zott, B. , Busche, M. A. , Sperling, R. A. , & Konnerth, A. (2018). What happens with the circuit in Alzheimer's disease in mice and humans? Annual Review of Neuroscience, 41, 277–297. 10.1146/annurev-neuro-080317-061725 PMC6571139 29986165

