# Supplementary material for: Hippocampal long‐term synaptic depression and memory deficits induced in early amyloidopathy are prevented by enhancing G‐protein‐gated inwardly rectifying potassium channel activity
Source: J Neurochem. 2020 Jan 30;153(3):362–76. doi: 10.1111/jnc.14946 (PMC7217154; doi:10.1111/jnc.14946)
Supplement: Supplementary file 1 [file JNC-153-362-s001.pdf]

Supplementary Material

Hippocampal long-term synaptic depression and memory deficits induced in early amyloidopathy are prevented by enhancing G-protein-gated inwardly-rectifying potassium channel activity

Irene Sánchez-Rodríguez[1*], Souhail Djebari[1*], Sara Temprano-Carazo[1*], David Vega-Avelaira[2], Raquel Jiménez-Herrera[1], Guillermo Iborra-Lázaro[1], Javier Yajeya[3], Lydia Jiménez-Díaz[1#] and Juan D. Navarro-López[1#]

[1]University of Castilla-La Mancha, NeuroPhysiology & Behavior Laboratory, Centro Regional de Investigaciones Biomédicas, School of Medicine of Ciudad Real, Spain.
[2]European University of Madrid, Departamento de Ciencias Biomédicas Básicas, Spain.
[3]University of Salamanca, Instituto de Neurociencias de Castilla y León, Salamanca, Spain.
*These authors contributed equally to this work.
[#]Contributed equally as corresponding authors.

Supplementary Figure 1.

I.c.v.-injected Aβ1-42 in freely moving mice primary reaches the dorsal hippocampus

To assess the degree of spread of intracerebroventricular (*i.c.v.*) injection of drugs in the brain of freely moving mice used in the present study, the extent of deposition of $A\beta_{1-42}$ peptide along the rostro-caudal axis was studied. Mice were injected with either vehicle or $A\beta_{1-42}$ through a guide cannula implanted chronically on the left ventricle (Sanchez-Rodriguez *et al.* 2017). Six i.c.v. injections were delivered in six consecutive days (1 injection per day) to maximize and assure clear results. Injections were performed in the morning timeframe (9 a.m. to 2 p.m.). One hour after the last injection animals were deeply anesthetized and perfused transcardially with saline and paraformaldehyde for further histological and immunohistochemical processing (material and methods described below). As shown in Fig. S1, immunohistochemical observations confirm that *i.c.v.* injection of $A\beta_{1-42}$ induces amyloidosis that primary affects the dorsal hippocampus.

Histology and Immunohistochemistry. Animals (n = 7) were deeply anesthetized 1 hour after the last injection with ketamine/xylazine administered intraperitoneally (75/10

mg/Kg; KETALAR®, Pfizer, Spain and ROMPUM®, Bayer, Spain), following the guidelines and authorization of the Ethical Committee of the University of Castilla-la Mancha due to their deep, stable and prolonged anesthetic effect. They received buprenorphine intramuscularly as analgesic (0.01mg/kg; # 062009, BUPRENODALE®, Albet, Spain). Mice were then perfused transcardially with 0.9% saline (#S9888, Sigma, Poole, UK) followed by 4% paraformaldehyde (#141451, Panreac Applichem, Barcelona, Spain) in phosphate-buffered saline (PBS; #P4417, Sigma, Poole, UK; 0.1 M, pH = 7.4). Their brains were removed and cryoprotected with 30% sucrose (#84100, Sigma, Poole, UK) in PB. Coronal sections (40 μm) were obtained with a sliding freezing microtome (Microm HM 450, Walldorf, Germany) and stored at −20°C in 50% glycerol (#G7757, Sigma, Poole, UK) in PBS until used. For immunoperoxidase staining, free-floating sections were pre-treated with 3% H_2O_2 (#31642-M, Sigma, Poole, UK) in methanol (#M/4000/17, Fisher Scientific, New Hampshire, US) for 30 seconds, and then immersed during 30 min in antigen retrieval buffer (#71405, sodium citrate; #71405, Sigma, Poole, UK; 10 mM pH = 6) heated up to 80ºC in a water bath. After washing with Tris-buffered saline (TBS; 2 x 10 min) and TBS containing 0.1% Triton X-100 (#T8532, Sigma, Poole, UK; TBS-T; 2 x 10 min), sections were treated for 45 min with 10% normal donkey serum (RRID:AB_2810235, Sigma, Poole, US) in TBS-T, and subsequently incubated overnight at room temperature with monoclonal mouse anti-Aβ primary antibody (1:500; RRID:AB_2564653, BioLegend, San Diego, US). The following day, sections were incubated for 2 hours at room temperature with 1:250 dilutions of biotinylated goat anti-mouse (RRID: AB_2340785, Jackson Immuno Research, West Grove, US), applying several washes with TBS-T before and after incubation with the secondary antibody. Then, sections were treated for 90 min with the avidin-biotin complex kit (RRID:AB_2336818, Vector Laboratories, Burlingame, US) and sequentially washed with TBS (2 x 5 min) and TB pH = 8 (2 x 5 min). Peroxidase activity was revealed by incubation in a solution containing 0.05% 3, 3'-diaminobenzidine (#D12384, Sigma, Poole, UK) as the chromogen, 0.01% H_2O_2 and nickel sulphate 1 mM (#A/5800/53, Fisher Scientific, New Hampshire, US) prepared in TB 0.1 M pH = 8. Afterwards, sections were thoroughly washed with TBS (3 x 10 min) and mounted on gelatin-coated glass slides. Lastly, sections were counterstained using the Nissl technique (with 0.25% Thionine; #8893, Sigma, Poole, UK), dehydrated and coverslipped using DPX (#44581, Sigma, Poole, UK). Staining specificity was checked by simultaneous incubation of negative controls (from vehicle-injected mice) and sections from A$\beta_{1\text{-}42}$-injected mice.

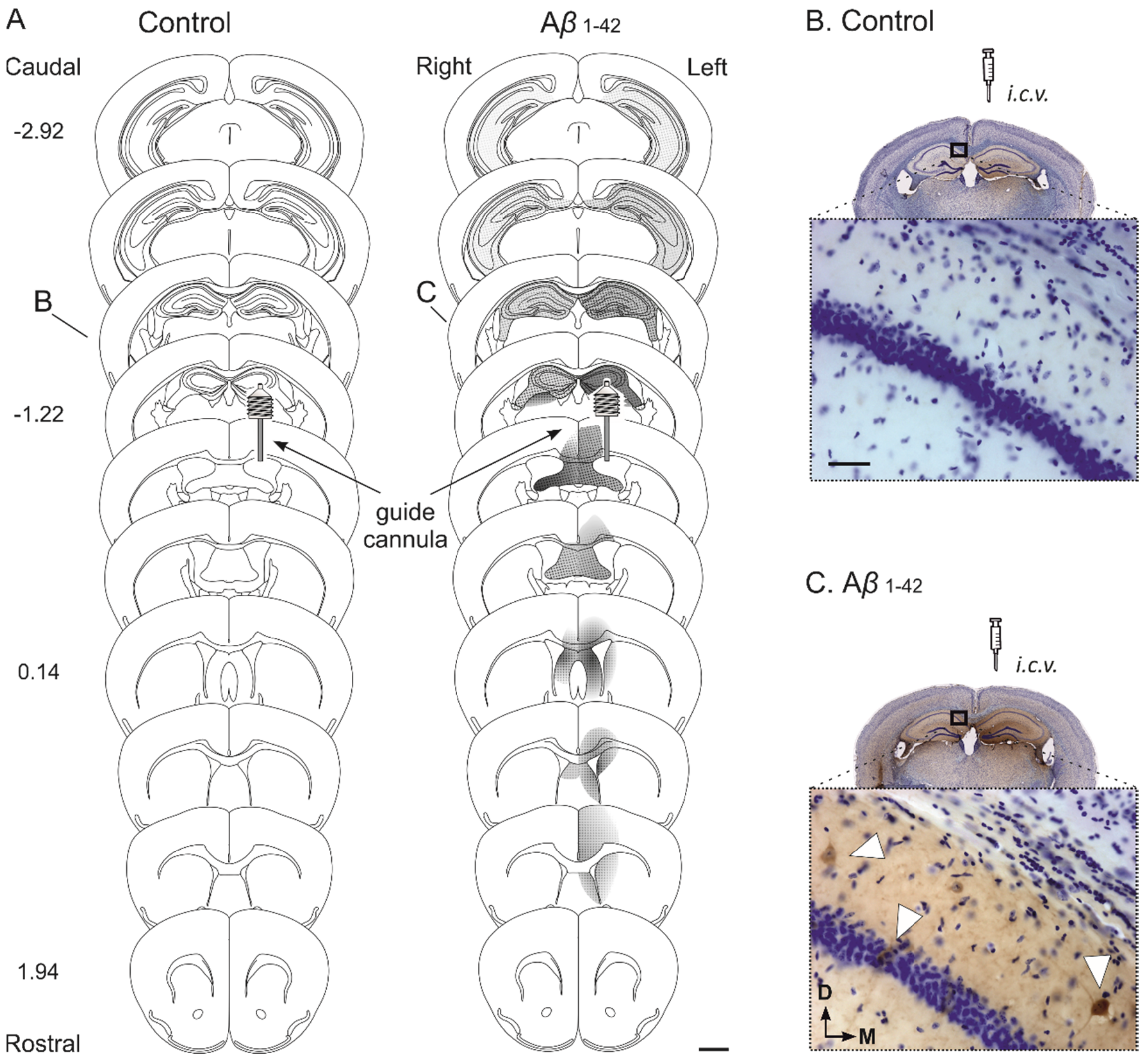

Figure S1. ***Aβ₁₋₄₂ deposition along the rostrocaudal axis of the hippocampus after i.c.v. injections.*** (A) Schematic representation of coronal brain sections arranged rostrocaudally from control (vehicle-injected, on the left of the panel) and $A\beta_{1\text{-}42}$-injected (on the right) mice. Rostrocaudal location of the implanted guide cannula is illustrated for both control and $A\beta_{1\text{-}42}$-injected mice. Shaded areas illustrate the location and degree of specific $A\beta_{1\text{-}42}$ staining and accumulation. Numbers on the left margin indicate the rostrocaudal distance in mm of the corresponding section from bregma. Sections represented in B and C are indicated in A with the corresponding capital letter. (B) Top: photomicrograph of a coronal brain section from a control mouse after immunoperoxidase staining against $A\beta_{1\text{-}42}$. Bottom: magnification of the CA1 area of the dorsal hippocampus is shown. (C) Top: photomicrograph of a coronal brain section from one $A\beta_{1\text{-}42}$-injected mouse. Bottom: magnification of the CA1 area of the dorsal hippocampus revealing intracellular deposition of $A\beta_{1\text{-}42}$ in neurons (white arrows). In B-C the hemisphere for *i.c.v.* injection is indicated. Squares in B-C indicate the magnified areas. Scale bar in A also applies for B and C (top low magnification sections), 1 mm; Scale bar in B (bottom picture) also applies for C (bottom picture), 40 µm. D, dorsal; M, medial.

Supplementary Figure 2.

Hippocampal GirK activation reverses habituation memory deficits induced by Aβ$_{1-42}$

Hippocampal-dependent open field habituation test. In mice, habituation to a novel environment (a non-associative hippocampal-dependent learning), is defined as a change in exploratory activity with repeated exposures (Leussis & Bolivar 2006). In the present study an open field habituation test was performed (re-exposure to an open field) to test this form of learning. Animals performed one trial per day in two consecutive days. Trial 1 on day 1 was the initial exposure to the open field (OF1 or training trial), and trial 2 was a 24 hours later re-exposure to the arena (OF2, retention or habituation trial). *I.c.v.* injections were performed 1 hour before retention trial. Either saline, Aβ$_{1-42}$, Aβ$_{1--42}$ + ML297, ML297, or Tertiapin-Q (TQ) were injected through a guide cannula implanted chronically on the left ventricle [1 mm lateral, 0.5 mm posterior to bregma, and 1.8 mm from the brain surface] (Sanchez-Rodriguez *et al.* 2017). The test was performed in an AC5 actimeter (Cibertec, Spain) consisting on an infrared system to detect animal movements in a square white plywood box (35 cm × 35 cm × 25 cm). On each trial, mice were placed at one of the four corners of the box and allowed to explore for 15 min. Total movements (X, Y, Z axis crossing summation) were recorded. The apparatus was cleaned with 70% ethanol to remove odors and allowed to dry before each animal was tested. Recorded data were analyzed with the MUX_XYZ16L software (Cibertec, Spain). Injections and behavioral tests were performed in the morning timeframe (9 a.m. to 2 p.m.).

In the training trial (OF1), there were no significant differences between travelled distances between mice assigned to each experimental group (Fig. S2B, C n = 36; $F_{(4,31)} = 1.2$, $p = 0.33$). On the habituation day (OF2: retention trial), exploratory movements were significantly reduced in all animals when compared to the training session (Fig. S2B, C), indicating they all remembered the open field arena (vehicle: $t(7) = 13.97$, $p = 2x10^{-6}$; Aβ: $t(4) = 7.02$; $p = 0.002$; Aβ + ML297: $t(4) = 8.48$; $p = 0.001$; ML297: $t(8) = 10.16$; $p = 8x10^{-6}$; TQ: $t(8) = 3.90$; $p = 0.005$). However, Aβ$_{1-42}$-injected animals explored significantly more than controls (vehicle-injected mice) during OF2 (Aβ$_{1-42}$ *vs.* vehicle: *Mann-Whitney* $U = 2$, $p = 0.008$), showing that Aβ$_{1-42}$ disrupted hippocampal-dependent habituation memory in these animals (Fig. S2B, C). The same was found for ML297- and TQ-injected mice and animals — i.e. both groups of animals explored more that vehicle control mice (ML297 *vs.* vehicle: *Mann-Whitney* $U = 4$, $p = 0.002$; TQ *vs.* vehicle: *Mann-Whitney* $U = 9$, $p = 0.009$) —, suggesting that both increasing or decreasing Girk channels activity by specific pharmacological modulation has a deleterious effect on hippocampal-dependent exploratory habituation. On the

contrary, level of habituation (measured as total exploration movements) of Aβ_{1-42} + ML297-treated mice did not differ to control (vehicle-injected) mice (Fig. S2B, C; Aβ_{1-42} + ML297 *vs.* vehicle: *Mann-Whitney* $U = 16$, $p = 0.558$) showing that these animals could recall the open field memory as well as control mice and highlighting the interest of GirK channels modulation in amyloidosis models.

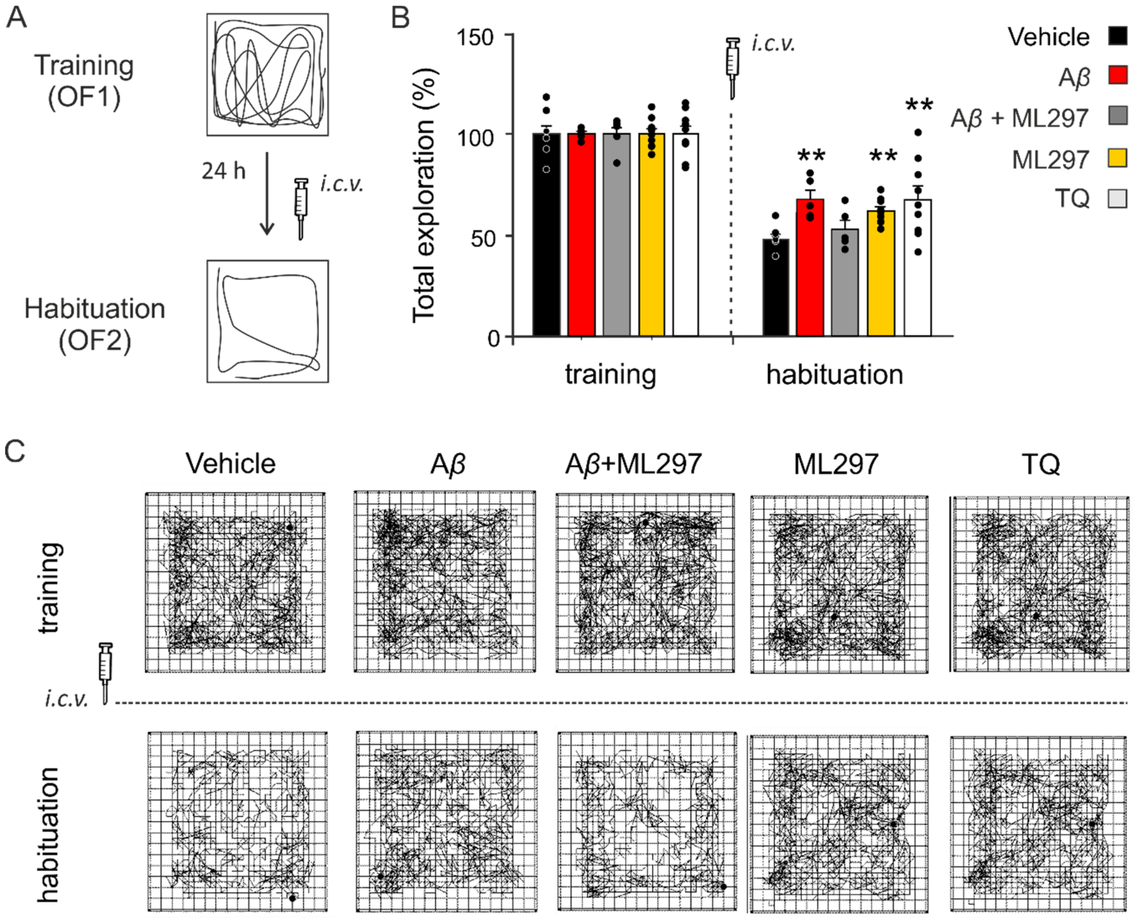

Figure S2. Effect of GirK modulation on mice with Aβ_{1-42}-induced amyloidosis in the dorsal hippocampus during hippocampal-dependent exploratory habituation. (A) Open field habituation test design. Mice were exposed for 15 min to the same open field two consecutive days with a 24 hours interval. Habituation memory levels were determined by measuring exploration behavior. *I.c.v.* injections were performed 1 hour before the retention trial (Open Field 2, OF2: habituation session). For each mouse, total exploration movements were tracked using an AC5 actimeter (Cibertec, Spain). Diagrams represent an example of the path followed by a control (vehicle-injected) animal during both training (OF1) and habituation (OF2) sessions. (B) Total exploration during 15 min-sessions for vehicle (n = 8 mice), Aβ_{1-42} (n = 5), Aβ_{1-42} + ML297 (n = 5), ML297 (n = 9), TQ (n = 9)-treated mice before (training) and after (habituation) drug administration. Data were normalized as percentage of the movement in the training session. Differences *vs.* control (vehicle) within OF2 session are indicated by asterisks crosses (**, $p < 0.01$). (C) Actimeter-generated tracking images illustrate the path followed by a representative animal of each experimental group during training and habituation sessions.

Supplementary Figure 3.

In vivo* and *in vitro drugs application did not affect spontaneous activity recorded from CA1 hippocampal region

In order to study the effect of the different drugs used in this work on hippocampal synchrony, we performed a spike analysis in local field potential recordings obtained from the CA1 region of the dorsal hippocampus both (Fig. S3A, C) Materials and methodology used to perform local field potential recordings and spike analysis are described below.

In vivo recordings (Fig. S3C, D) were obtained immediately before and 24 hours after *i.c.v.* injections of vehicle (n = 9 animals), Aβ_{1-42} (n = 6), the combination Aβ_{1-42} + ML297 (n = 7), ML297 (n = 10), or TQ (n = 6), and the total number of spikes was quantified. Animals used in this study did not show any form of epileptiform activity before or after injections. The spikes we analyzed were small in amplitude and duration (~1 ms), and we believe them to be an indicative of higher levels of synchronous activity in the hippocampal neural network. The number of spikes after drug administration was normalized as percentage of pre-injection values. Mice injected with vehicle presented a similar number of spikes before and after injections (Fig. S3D, $t(8) = 0.44$, $p = 0.66$). In the other groups, there were also no significant differences with pre-injection values (dotted line, Fig. S3D), indicating that drugs did not modify spike number. There were also no significant differences between experimental groups ($F_{(4,33)} = 0.45$, $p = 0.995$).

In vitro spike count after hippocampal slice treatment with the same drugs describe above (vehicle, Aβ_{1-42}, Aβ_{1-42} + ML297, ML297, or TQ; n = 5 slices for each group) showed similar results (Fig. S3C, D), with no differences in synchrony between treatments after 10 min of drug administration ($F_{(3,16)} = 0.935$, $p = 0.447$). Taken together, these results show that the acute administration of the drugs used in this study do not produce significant alterations in the hippocampal network synchrony.

Spike analysis in local field potentials recordings in vivo and in vitro.

Local field potential recording <u>*in vivo*</u>. Surgery for chronic recordings and *i.c.v.* injections followed in the present study have been previously described (Sanchez-Rodriguez *et al.* 2017). Briefly, subjects were anesthetized with ketamine/xylazine administered intraperitoneally (75/10 mg/Kg; KETALAR®, Pfizer, Spain and ROMPUM®, Bayer, Spain), following the guidelines of the Ethical Committee of the University of Castilla-la Mancha due to their deep and prolonged anesthetic effect. They received buprenorphine intramuscularly as analgesic (0.01mg/kg; # 062009, BUPRENODALE®, Albet, Spain). Animals were then implanted with stimulating

electrodes aimed at the right Schaffer collateral-commissural pathway of the dorsal hippocampus (2 mm lateral and 1.5 mm posterior to bregma; depth from brain surface, 1.0–1.5 mm), and with recording electrodes aimed at the ipsilateral *stratum radiatum* underneath the CA1 area (1.2 mm lateral and 2.2 mm posterior to bregma; depth from brain surface, 1.0–1.5 mm). The final position of the hippocampal electrodes was determined by evaluating the field potential depth profile evoked responses presented at the Schaffer collateral pathway. A bare silver wire (0.1 mm) was affixed to the skull as a ground. All electrodes and the ground were connected to a 6-pin socket that was fixed to the skull with dental cement. Animals were also implanted chronically with a blunted, stainless steel, 26-G guide cannula (Plastic One, Roanoke, VA, USA) in the contralateral ventricle (0.5 mm posterior to bregma, 1.0 mm lateral to midline, and 1.8 mm below the brain surface), in order to perform the *i.c.v.* administration of drugs included in this study and preserve the contralateral CA3–CA1 pathway intact to perform electrophysiological recordings in alert mice. Mice were allowed a week for recovery before the experimental sessions. Local field potentials were recorded with Grass P511 differential amplifiers through a high-impedance probe (2 × 1012 Ω, 10 pF) from hippocampal CA1 region, for 5 min in total absence of electrical stimulation, while animals were alert and freely moving inside a small box (5 × 5 × 5 cm). Recordings were acquired twice: before (pre-injection values) and 24 hours after *i.c.v* injections. Injections and recordings were performed in the morning timeframe (9 a.m. to 2 p.m.).

Local field potential recording in vitro. Same type of recordings as *in vivo* —i.e. local field potentials in total absence of electrical stimulation —were collected from the CA1 area of hippocampal slices *in vitro*, for 10 min in aCSF solution (vehicle/control) and then for 10 min after drug administration into the bath chamber. Each whole recording was processed in Spike2 (CED, Cambridge, UK) with a fourth order IIR high pass filter (corner = 300) and used for spike quantification.

Spike analysis. For both *in vivo* and *in vitro* recordings, an event with an amplitude exceeding 4X the standard deviation of an event-free timeframe of the recording amplitude was considered to be a spike (Fig. S3B). The total number of spikes was represented as a percentage of control (*in vivo* control: pre-injection; *in vitro* control: aCSF vehicle) values.

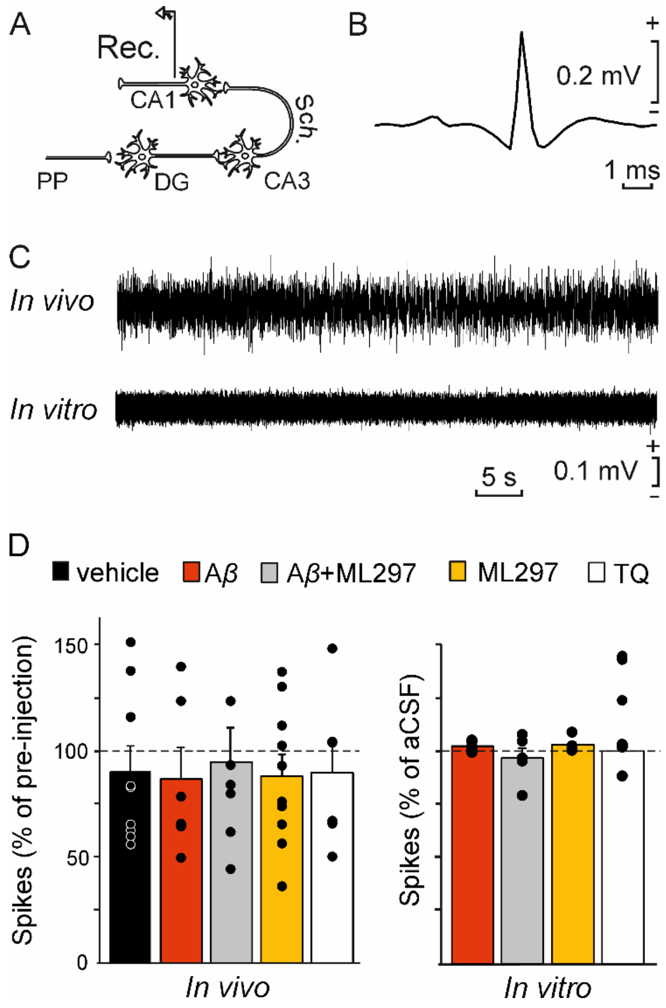

Figure S3. Spike analysis in local field potentials recordings in vivo and in vitro.
(A) Schematic representation of the location of the recording electrode in the CA1 area of the dorsal hippocampus for local field potential (LFP) recordings. (B) Representative example of averaged ($n = 5x10^5$) spikes that illustrates the spikes profile analyzed in the LFP recordings. (C) Representative examples of LFPs recordings from the CA1 area *in vivo* and *in vitro*. (D) On the left: *in vivo* LFPs were recorded from the CA1 area in alert freely moving animals and used for spike analysis. Events with a peak-to-peak amplitude higher than four standard deviations from the LFP background were considered spikes. The total number of spikes present on a 5 min LFP recording was quantified before (baseline) and 24 hours after *i.c.v.* injections of vehicle (n = 9), $A\beta_{1-42}$ (n = 6), $A\beta_{1-42}$ + ML297 (n = 7), ML297 (n = 10), or TQ (n = 6). Bars represent spike number, normalized as percentage of pre-injection values (100%, dotted line). There were no significant differences between experimental groups after injections *in vivo* ($p > 0.05$). On the right, *in vitro* extracellular recordings were acquired from the CA1 hippocampal region of mouse brain slices. Bars represent spike number after bath application of $A\beta_{1-42}$, $A\beta_{1-42}$ + ML297, ML297, or TQ (n = 5 slices for each group, normalized as a percentage of spikes before drug perfusion (i.e. slices inmersed in aCSF (vehicle); 100%, dotted line). There were no significant changes in spike density in any experimental group when compared to baseline values *in vitro* ($p > 0.05$). Rec, recording electrode; Sch., Schaffer collateral pathway; PP, Perforant pathway; DG, dentate gyrus; aCSF, artificial cerebrospinal fluid.

Supplementary Figure 4

I.c.v. drugs application did not affect motor activity

Motor activity was determined by a rotarod test (Kaufmann *et al.* 2013). The rotarod apparatus (Harvard Apparatus, Holliston, MA, US) consisted of a black striated rotating rod (3 cm in diameter) 20 cm above the floor, set to accelerate from 4 to 40 rpm over a 5 min period. First, animals got used to the apparatus by walking on the rod with constant low-speed rotation (10 rpm) for 1 min. The test was started afterwards. Time until mice fell from the rod ("latency") was measured automatically. After *i.c.v.* injections, mice (vehicle, n = 7; A$\beta_{1\text{-}42}$, n = 8; A$\beta_{1\text{-}42}$ + ML297, n = 7; ML297, n = 6; or TQ, n = 7) were tested for one session consisting of 6 trials separated by 5 min resting periods. Average of latencies of the 6 trials was calculated. Injections and behavioral tests were performed in the morning timeframe (9 a.m. to 2 p.m.). As shown in Fig. S4, no differences were found between experimental groups when considering the whole session (Fig. S4A; $F_{(4,30)} = 0.53$, $p = 0.994$), or when individual trials were analyzed (Fig. S4B; $F_{(4,30)} = 90.85$, $p = 0.954$). All animals improved their performance along trials with no differences between groups (*Greenhouse-Geisser correction*, $F_{(2.22,66.7)} = 0.16$, $p < 0.001$; repeated-measures two-way ANOVA was conducted to examine the main effect of treatment on each trial. *Greenhouse-Geisser* correction was used as sphericity was not assumed).

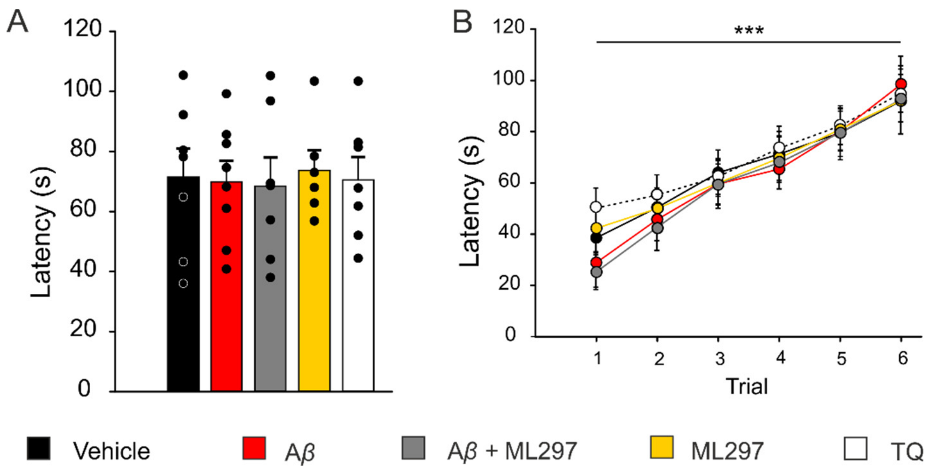

Figure S4. ***I.c.v. drugs application did not affect motor coordination.***
(A-B) Latency to fall of mice *i.c.v.*-injected with vehicle (n = 7), A$\beta_{1\text{-}42}$ (n = 8), A$\beta_{1\text{-}42}$ + ML297 (n = 7), ML297 (n = 6), or TQ (n = 7) during testing on the accelerated rotarod. Sessions consisted of 6 trials separated by 5 min resting periods. Mean latency corresponding to the whole session (A, bars) or to each testing trial (B) are illustrated. No differences were found between experimental groups when considering the whole session (Fig. S4A; $F_{(4,30)} = 0.53$, $p = 0.994$), or when individual trials were analyzed (Fig. S4B; $F_{(4,30)} = 90.85$, $p = 0.954$). All animals showed similar improvement rate along trials regardless the treatment received (*Greenhouse-Geisser correction*, $F_{(2.22,66.7)} = 0.16$, $p < 0.001$). ***, $p < 0.001$.

Reference List

Kaufmann, K., Romaine, I., Days, E. et al. (2013) ML297 (VU0456810), the first potent and selective activator of the GIRK potassium channel, displays antiepileptic properties in mice. *ACS Chem. Neurosci.* **4,** 1278-1286.

Leussis, M. P. and Bolivar, V. J. (2006) Habituation in rodents: a review of behavior, neurobiology, and genetics. *Neurosci Biobehav Rev* **30,** 1045-1064.

Sanchez-Rodriguez, I., Temprano-Carazo, S., Najera, A., Djebari, S., Yajeya, J., Gruart, A., Delgado-Garcia, J. M., Jimenez-Diaz, L. and Navarro-Lopez, J. D. (2017) Activation of G-protein-gated inwardly rectifying potassium (Kir3/GirK) channels rescues hippocampal functions in a mouse model of early amyloid-beta pathology. *Sci Rep* **7,** 14658.
