# Supplementary material for: Hippocampal long‐term synaptic depression and memory deficits induced in early amyloidopathy are prevented by enhancing G‐protein‐gated inwardly rectifying potassium channel activity
Source: J Neurochem. 2020 Jan 30;153(3):362–76. doi: 10.1111/jnc.14946 (PMC7217154; doi:10.1111/jnc.14946)
Supplement: Supplementary file 2 [file JNC-153-362-s002.pdf]

Open Practices Disclosure

Manuscript Title: Hippocampal long-term synaptic depression and memory deficits induced in early amyloidopathy are prevented by enhancing G-protein-gated inwardly-rectifying potassium channel activity
Corresponding Author: Juan D. Navarro López and Lydia Jimenez Díaz

Articles accepted to *Journal of Neurochemistry* after 01.2018 are eligible to earn badges that recognize open scientific practices: publicly available data, material, or preregistered research plans. Please read more about the badges in our *author guidelines and Open Science Badges page*, and you can also find information on the Open Science Framework wiki.

[X] Please check this box if you are interested in participating.

To apply for one or more badges acknowledging open practices, please check the box(es) corresponding to the desired badge(s) below and provide the information requested in the relevant sections. To qualify for a badge, you must provide a URL, doi, or other permanent path for accessing the specified information in a public, open-access repository. **Qualifying public, open-access repositories are committed to preserving data, materials, and/or registered analysis plans and keeping them publicly accessible via the web in perpetuity.** Examples include the Open Science Framework (OSF) and the various Dataverse networks. Hundreds of other qualifying data/materials repositories are listed at http://re3data.org/. Preregistration of an analysis plan must take place via a publicly accessible registry system (e.g., OSF, ClinicalTrials.gov or other trial registries in the WHO Registry Network, institutional registration systems). **Personal websites and most departmental websites do not qualify as repositories.**

Authors who wish to publicly post third-party material in their data, materials, or preregistration plan must have the proper authority or permission agreement in order to do so.

There are circumstances in which it is not possible or advisable to share any or all data, materials, or a research plan publicly. For example, there are cases in which sharing participants' data could violate confidentiality. If you would like your article to include an explanation of such circumstances and/or provide links to any data or materials you have made available—even if not under conditions eligible to earn a badge—you may write an alternative note that will be published in a note in the article**.** Please check this box if you would like your article to include an alternative note and provide the text of the note below:

[] Alternative note:

[] Open Data Badge

1. Provide the URL, doi, or other **permanent path** for accessing the data in a **public, open-access repository**:

[] Confirm that there is sufficient information for an independent researcher to reproduce **all of the reported results**, including codebook if relevant.

[] Confirm that you have registered the uploaded files so that they are **time stamped** and cannot be age.

[] Open Materials Badge

1. Provide the URL, doi, or other **permanent path** for accessing the materials in a **public, open-access repository**: all relevant information is provided in the manuscript and custom-made materials will be provided upon reasonable request.

[X] Confirm that there is sufficient information for an independent researcher to reproduce **all of the reported methodology**.

[] Confirm that you have registered the uploaded files so that they are **time stamped** and cannot be age.

[] Preregistered Badge

1. Provide the URL, doi, or other **permanent path** to the registration in a **public, open-access repository***:

2. Was the analysis plan registered prior to examination of the data or observing the outcomes? If no, explain.**

3. Were there additional registrations for the study other than the one reported? If yes, provide links and explain.*

*No badge will be awarded if (1) is not provided, **or** if (3) is answered “yes” without strong justification

**If the answer to (2) is “no,” the notation DE (Data Exist) will be added to the badge, indicating that registration postdates realization of the outcomes but predates analysis.

By signing below, authors affirm that the above information is accurate and complete, that any third-party material has been reproduced or otherwise made available only with the permission of the original author or copyright holder, and that publicly posted data do not contain information that would allow individuals to be identified without consent.

Date: ______________________

Name: ______________________

Signature: ______________________

JIMENEZ DIAZ LYDIA - 44297228X

Firmado digitalmente por JIMENEZ DIAZ LYDIA - 44297228X
Nombre de reconocimiento (DN): c=ES, serialNumber=IDCES-44297228X, givenName=LYDIA, sn=JIMENEZ DIAZ, cn=JIMENEZ DIAZ LYDIA - 44297228X
Fecha: 2019.12.06 14:38:07 +01'00'

NAVARRO LOPEZ JUAN DE DIOS - 44295425R

Firmado digitalmente por NAVARRO LOPEZ JUAN DE DIOS - 44295425R
Nombre de reconocimiento (DN): c=ES, serialNumber=IDCES-44295425R, givenName=JUAN DE DIOS, sn=NAVARRO LOPEZ, cn=NAVARRO LOPEZ JUAN DE DIOS - 44295425R
Fecha: 2019.12.06 14:38:50 +01'00'
